# Do mindfulness-based interventions change brain function in people with substance dependence? A systematic review of the fMRI evidence

**DOI:** 10.1186/s12888-023-04789-7

**Published:** 2023-06-07

**Authors:** Valentina Lorenzetti, Alexandra Gaillard, Emillie Beyer, Magdalena Kowalczyk, Sunjeev K. Kamboj, Victoria Manning, John Gleeson

**Affiliations:** 1 Neuroscience of Addiction and Mental Health Program, Healthy Brain and Mind Research Centre, School of Behavioural and Health Sciences, Faculty of Health Sciences, Australian Catholic University, Level 5 Daniel Mannix Building, 115 Victoria Parade, Fitzroy, VIC 3065 Australia; 2 Centre for Mental Health, Swinburne University of Technology, Hawthorn, Australia; 3 Research Department of Clinical, Educational and Health Psychology, University College London, London, UK; 4 Monash Addiction Research Centre, Eastern Health Clinical School, Monash University, Melbourne, Australia; 5 Turning Point, Eastern Health, Monash University, Melbourne, Australia; 6 Digital Innovations in Mental Health and Well-being Program, Healthy Brain and Mind Research Centre, School of Behavioural and Health Sciences, Australian Catholic University, Melbourne, VIC Australia

**Keywords:** Substance use disorder (SUD), Addiction, Mindfulness, Mindfulness-based intervention (MBI), Functional magnetic resonance imaging (fMRI), Review

## Abstract

**Background:**

Substance use disorders (SUDs) affect ~ 35 million people globally and are associated with strong cravings, stress, and brain alterations. Mindfulness-based interventions (MBIs) can mitigate the adverse psychosocial outcomes of SUDs, but the underlying neurobiology is unclear. Emerging findings were systematically synthesised from fMRI studies about MBI-associated changes in brain function in SUDs and their associations with mindfulness, drug quantity, and craving.

**Methods:**

PsycINFO, Medline, CINAHL, PubMed, Scopus, and Web of Science were searched. Seven studies met inclusion criteria.

**Results:**

Group by time effects indicated that MBIs in SUDs (6 tobacco and 1 opioid) were associated with changes in the function of brain pathways implicated in mindfulness and addiction (e.g., anterior cingulate cortex and striatum), which correlated with greater mindfulness, lower craving and drug quantity.

**Conclusions:**

The evidence for fMRI-related changes with MBI in SUD is currently limited. More fMRI studies are required to identify how MBIs mitigate and facilitate recovery from aberrant brain functioning in SUDs.

**Supplementary Information:**

The online version contains supplementary material available at 10.1186/s12888-023-04789-7.

## Introduction

Substance use disorders (SUDs) affected ~ 36 million people aged 15-to-64 over the past year globally [1], and costs more than U$600 billion annually in the US alone [2]. SUDs can have significant negative neurobiological and psychosocial outcomes. They include: brain dysfunction in pathways implicated in reward and salience processing, motivation and disinhibition; intense cravings, and hazardous behaviors (e.g., operating machinery while intoxicated), compulsive use despite the experience of significant psychosocial harms; mental health problems (e.g., depression, anxiety, psychoses) [3–5]; as well as high relapse rates within the first year of treatment (i.e., 40%-to-60%) [2]. These negative impacts are alarming and warrant the development of new effective therapies including those that directly target the core neurobiological mechanisms of SUD, in keeping with recent calls to develop new and effective treatments (e.g., National Institute of Drug Abuse Strategic Plan [6]).

In recent years, psychological therapies have incorporated mindfulness. As a psychological faculty, ‘mindfulness’ has been defined as ‘awareness that arises through paying attention, on purpose, in the present moment, non-judgmentally’ [7]. Treatments that are designed to encourage individuals to develop this mindfulness capability and incorporate it into daily life to improve emotional, behavioral, and cognitive outcomes—Mindfulness based interventions (MBIs)—have targeted several mental health problems that often co-occur with SUD (e.g., anxiety, stress) [8]. Meta-analyses in healthy control samples established that MBIs boost cognitive control, mitigate stress reactivity (dysfunction of which is also associated with SUDs) and that MBIs target and change the function of neural pathways underlying such processes (e.g., cingulate, prefronto-parietal and cerebellar regions, insula and cerebellum) [9–14].

MBIs have been adapted to target specific aspects of SUD: boosting top-down cognitive control over habitual behavior, increasing attention and responsivity to natural rewards [15, 16], and diminishing stress reactivity and craving [15, 17, 18]. Meta-analyses show that MBIs have clinical efficacy in treating SUDs in relation to distinct substances and behaviors e.g., tobacco, alcohol, opiates, cocaine, stimulants, cannabis, gaming, as well as polysubstance use [2, 15, 18]. Meta-analyses have revealed MBIs can decrease SUD-relevant outcomes, compared to alternative and/or control interventions [15, 17, 18, 20]. Notably, control interventions varied somewhat e.g., no intervention, support group, treatment as usual, relapse prevention, freedom from smoking, coping as usual, National Cancer Institute’s QuitGuide App, and others [15, 17, 18, 20]. SUD-related outcomes decreased by MBIs include (but are not limited to), reduced severity of craving/withdrawal will people know (i.e., SMD from − 0.19 to − 0.07 [17], and SMD from − 0.11 to − 0.25 [18]), reduced stress (i.e., SMD from − 2.24 to − 0.01 [18]), negative consequences of substance use (i.e., SMD from − 0.45 to − 0.01) [17], lower depression symptom scores (i.e., SMD − 0.49 to 0.32) [17], and increased mindfulness (i.e., SMD − 1.35 to 0.78) [18] when contrasted with alternative and/or control treatments.

The benefits of MBIs for people with a SUD have been ascribed to the targeting of the function (i.e., activity, connectivity) of a ‘mindfulness network’ – with specific inhibitory, attentional/salience and stress related brain pathways, which MBIs have been shown to target in healthy controls. Specifically, MBIs can boost the function of areas within this network (e.g., prefrontal, anterior cingulate cortex [ACC], and parietal cortices) [21–23]. Notably, the function of such disinhibitory and salience pathways is altered in SUDs [4]. Alteration of such pathways is also postulated to reflect the core pathophysiology of SUDs [4], and therefore their integrity should be restored via means of interventions in order to mitigate SUD-related adverse outcomes. Of relevance, MBIs have been shown to down-regulate regions implicated in stress in healthy samples [15]. Notably, stress and reward related pathways in substance users are also implicated in craving and reactivity to drug-related cues (e.g., striatum, amygdala). However, the emerging fMRI evidence on how MBIs affect brain function in SUDs are yet to be systematically synthesized. Therefore, it is unclear whether the treatment effect associated with MBIs for SUDs correlate with neural changes as shown in healthy samples [21–23], and whether changes in brain function that occur with MBIs, are associated with increased state or trait mindfulness, lower substance use and cravings and other behavioral indices of problematic drug use.

Integrating the literature on the neurobiological changes associated with MBIs in SUDs (e.g., opiates, nicotine) has significant potential benefits. Such knowledge could have implications for informing the development of neuroscientific theories of MBI and SUD. Further, brain function can predict relapse and changes in psychological states relevant for relapse such as craving and stress [4]. Therefore, if MBIs were effective in restoring the altered neurobiology within SUD-related brain pathways implicated in dysfunctional reward processing, craving and stress—in addition to changing these negative psychological states—this new knowledge might inform the development of more effective MBIs that engage both neural mechanisms and behavioral outcomes of SUD with longer lasting effects.

Our primary aim in the current review was to systematically integrate the fMRI evidence for brain functional changes associated with MBIs in SUDs. Our secondary aim was to summarize the evidence of correlations between brain functional changes associated with MBI, state or trait or both mindfulness levels, proximal indices of problematic substance use (i.e., craving and quantity used) and other behavioral, wellbeing and subjective measures associated with drug use (e.g., positive affect, impulsivity). Finally, we aimed to systematically assess the quality of the MBIs and fMRI methodologies in the reviewed studies as a preliminary basis for recommending minimum methodological standards for future fMRI studies in this area. Such standards will enable greater precision in identifying the neural mechanisms underlying the treatment effects of MBIs for SUDs, ultimately informing the identification of neurobiological targets for MBIs that target craving and stress in order to reduce or eliminate problematic substance use.

## Methods

### Protocol and Registration

This systematic review was conducted following the recommended principles of the Centre for Reviews and Dissemination [24]. The search strategy was reported according to the Preferred Reporting Items for Systematic Reviews and Meta-Analyses guidelines (PRISMA) [25] (see Supplementary Table 1). The study protocol was developed and registered on PROSPERO (submitted 26/04/2021 and approved 30/05/2021; ID CRD42021242545).

### Literature search

A comprehensive literature search with no time limit, was conducted using PsycINFO (EBSCOhost), MEDLINE (EBSCOhost), CINAHL (EBSCOhost), Web of Science, Scopus, and PubMed on April 26, 2021. The search strategy used three concepts related to (i) substance use, (ii) mindfulness-based interventions, and (iii) functional neuroimaging. Medical Subject Headings (MeSH), text, and keywords were combined with Boolean OR/AND operators. Search terms per database are outlined across Supplementary Tables 2–7.

### Study selection

The PRISMA flowchart, summarizing the screening process for all included studies which was carried out using Covidence (www.covidence.org) can be found in Supplementary Fig. 1. The searches retrieved 735 articles. A researcher (E.B) screened all retrieved articles’ title and abstracts and then reviewed the full text to determined data extraction eligibility. A second researcher (A.G.) additionally confirmed studies selection. After removal of duplicates, the titles and abstracts of **451 articles** were screened against selected inclusion and exclusion criteria. *Inclusion criteria* were: (i) human participants, (ii) sample comprising regular substance users as defined by each study; (iii) use of fMRI to measure brain function, (iv) administration of an MBI intervention and mindful practices; (v) English language publication; (vi) peer reviewed. *Exclusion criteria* were: (i) non-human sample; (ii) studies where all participants had confirmed dual diagnosis of substance use and a mental health disorders (e.g., major depressive disorder, anxiety disorder, psychotic disorder); (iii) neurological disorders and major medical conditions that affect the central nervous system (e.g., HIV); (iv) multicomponent intervention that included mindfulness (e.g., acceptance and commitment therapy); (v) use of neuroimaging techniques other than fMRI (e.g., diffusion tensor imaging) and MRI (e.g., EEG, Positron Emission Tomography, SPECT); (vi) Brain function measured during acute substance intoxication; (vii) non-published and non-peer-reviewed (e.g., conference abstracts, dissertations); (viii) non-empirical studies (e.g., single case reports, book chapters, letters to the editor, reviews). Finally, **15 articles** were retained for full-text review against inclusion and exclusion criteria. The reference lists of selected studies were cross-referenced for additional work that was relevant for the review.

### Data extraction

Data extraction was conducted during screening of full-text articles. First, we extracted data on the details of each publication (e.g., first author, year, location), demographic and substance use characteristics of each sample (e.g., age and sex, substance type, duration and quantity; Table 1). Second, we summarized data on the parameters of the interventions (e.g., type, duration, mode of delivery; see Supplementary Table 8) and on the timing of the fMRI or MBI in relation to assessment timepoints (see Supplementary Table 9). Third, we summarized data on the fMRI parameters used, including the fMRI task type (e.g., resting-state, task-based), analysis approach (e.g., whole brain, seed-based) and property of brain function examined (e.g., activity, connectivity; Tables 2 and 3; Supplementary Table 10). We also noted missing data and accounted for this information in the risk of bias assessment with all relevant summaries and interpretation of results (details on the risk of bias assessments can be found in *Supplementary Text 3*, Supplementary Tables 11–12).

**Table 1:** Overview of sample demographic and substance use characteristics

Author, year	N total(female)		Age, mean		Substance exposure at baseline			MRI assessment
	MBI	Control	MBI	Control	Type	Duration (years)	Quantity	
Fahmy, Wasfi (26)	16(1)	16(1)	30	32	Opiates	10	-	Baseline + Follow-up
Janes, Datko (28)	33(20)	34(25)	46	43	Nicotine	-	17 cig/day	Baseline + Follow-up
Kragel, Sweitzer (30)	5(2)	-	18+	-	Nicotine	17	≥10 cig/day	Baseline + Follow-up
Froeliger, Mathew (27)	7(3)	6(1)	50	48	Nicotine	25	22 cig/day	Baseline + Follow-up
Kober, Brewer (29)	11(4)	12(3)	48	49	Nicotine	-	18 cig/day	Follow-up
Tang, Tang (9)	Smokers: 14(4) Non-smoker: 18+	Smokers: 11(4) Non-smoker: 15	22	22	Nicotine	-	10 cig/day	Baseline + Follow-up
Westbrook, Creswell (2)	54(37)	-	45	-	Nicotine	26	18 cig/day	Single scan

Abbreviations: MBI, Mindfulness−based intervention; N, Number of participants; cig; cigarettes

**Table 2:** Overview of neurobehavioral *intervention-by-time* effects in substance use disorders

Author, Year	Behavior changes pre-post MBI vs. control		fMRI			Brain changes pre-to-post MBI vs. control intervention				Brain-behavior associations
	Substance use	Mindfulness/other	Task	Analysis	Function	Direction, region	Cohen’s *d*	*p*, correction	Z or t	
Fahmy, Wasfi (26)			Rest (eyes closed)	Whole brain ICA (DMN)	Connectivity	↓ IFG (right, aDMN)		P < .05 FDR-corrected	Z = 4.72	Collapsed groups: *Neg. Cor.* FMI & rIFG connectivity
Janes, Datko (28)	**=** Cigs/day		Cue reactivity (nicotine vs. neutral)	ROI (PCC)	Activity	= PCC cue-reactivity	0.02	P < .05 permutation -corrected		
Froeliger, Mathew (27)	↓ Cigs/week = Craving & urge to smoke change (SJWQ), breath carbon monoxide	↑ positive affect (PANAS) = negative affect (PANAS), affect (VAS)	Emotion (positive vs. neutral)	Whole brain	Activity	↑ S*triatum* (VS/caudate), ACC (rostral/vmPFC)	2.13–2.66	P < .05 montecarlo -corrected	Z = 2.9–3.5	Collapsed groups: *Pos. Cor.* Magnitude smoking reduction, ventral striatum & rACC *Neg. Cor.* Urge to smoke & ventral striatum *N.S. Cor.* trend urge to smoke & rACC *Pos. Cor.* positive affect & rACC *N.S. Cor.* trend observed for positive affect & ventral striatum
			Cue reactivity (nicotine vs. neutral)	Whole brain	Activity	↓ S*triatum* (VS/caudate), ACC (rostral/vmPFC)	1.57–1.7		Z = 2.1–2.3	
			Seeds from emotion & cue reactivity tasks	Seed-whole brain (rACC)	Connectivity	↑ ACC (rostral OFC)	2.69		F = 19.8	Collapsed groups: *Pos. Cor.* Magnitude smoking reduction, positive affect & rACC-OFC connectivity
Kober, Brewer (29)^*^	↓ Cigs/day (group*time) = Craving (VAS)	= Stress (VAS)	Stress reactivity (stress vs. neutral – verbal scenarios)	Whole brain	Activity	↓ S*triatum* (putamen), cerebellum, insula (ant/mid/post), amygdala, hippocampus, para-hippocampus, thalamus, midbrain	1.03–1.10	P < .05 FWE-corrected	t = 3.97–5.72	*MT*: *Pos. Cor.* Cigs/day & insula (ant), amygdala, para-hippocampus *FFS*: *N.S. Cor.* Cigs/day & insula (ant), amygdala, para-hippocampus
						Insula (ant), amygdala, para-hippocampus				Combined groups: *Pos. Cor.* Cigs/day reduction & activity (amygdala, ant/mid/post insula, hippocampus, para-hippocampus, thalamus, mid occipital, midbrain, cerebellum, cuneus/precuneus, PCC)
Tang, Tang (9)^*^	= Craving (group*time) ↓ carbon monoxide (group*time)		Rest (eyes closed)	Whole brain (fALFF)	Activity	↓ Cerebellum, precuneus/PCC/BA31		*P < .05* montecarlo -corrected	*t* = (-3.91)-(-4.10)	
Westbrook, Creswell (2)*	↓ Craving (VAS)	↓ stress (VAS)	Cue reactivity (neutral vs. cigarette)	Whole brain	Activity	*Passive view cigs > neutral*: ↑ ACC (ventral/mFG), precuneus ↓ fusiform *Mindful > passive view cigs*: ↓ ACC (subgenual/vmPFC)		*P < .05* montecarlo -corrected	*t* = 3.58–4.90	
				Seed-whole brain (sgACC) PPI	Connectivity	*Mindful > passive view cigs*: ↓ Striatum (VS/caudate), precuneus, insula, MFG, parietal (inferior), premotor			*t* = (-4.15)–(-5.38)	

*Groups compared cross−sectionally, post intervention

↑ Increased; ↓ Decreased; *=* no change

Abbreviations: ACC, anterior cingulate cortex; aDMN, anterior default mode network; Ant, anterior; BA, Brodmann area; CO, carbon monoxide; Cor, correlation; DTS, distress tolerance scales; fALFF, fractional amplitude of low−frequency fluctuations; FFS, freedom from smoking; FMI, Freiburg mindfulness inventory; FTND, Fagerstrom test for nicotine dependence; ICA, independent component analysis; IFG, inferior frontal gyrus; MBI, Mindfulness−based intervention; MFG, middle frontal gyrus ; mFG, medial frontal gyrus; N.S., non−significant; Neg, negative; OFC, orbitofrontal cortex; PANAS, positive and negative affect schedule; PCC, posterior cingulate cortex; pDMN, posterior default mode network; PPI, psychophysiological interactions; Pos, positive; Post, posterior; rACC, rostral anterior cingulate cortex; ROI, region of interest; sgACC, subgenual anterior cingulate cortex; SJWQ, Shiffman−Jarvik questionnaire; TAU, treatment as usual; UPPS, impulsive behavior scale; VAS, visual analogue scale; vmPFC, ventromedial prefrontal cortex; VS, ventral striatum

**Table 3:** Overview of neurobehavioral changes *pre-to-post MBI* in substance use disorder

Author, Year	Behavior changes pre-post *mindfulness* intervention		fMRI			Brain functional changes				Brain-behavior associations
	Substance use	Mindfulness/other	Task	Analysis method	Function	Direction, region	Cohen’s *d*	*p*, correction	Z or t	
Fahmy, Wasfi (26)		↑ Mindfulness (FMI), stress tolerance/appraisal/ absorption/regulation (DTS) ↓ negative urgency (UPPS-P) = positive urgency, premeditation, perseverance, sensation seeking (UPPS-P)	Rest (eyes closed)	Whole brain ICA (aDMN, pDMN)	Connectivity	↓ rSFG (aDMN) = pDMN		*P < .05* FDR-corrected	Z = 4	*Neg. Cor.* Mindfulness (FMI) & rSFG connectivity r = − .6 * N.S. Cor.* UPPS-P (negative urgency, premeditation, perseverance), DTS (tolerance, appraisal, absorption, regulation) & rSFG connectivity
Janes, Datko (28)	↓ Cigs/day		Cue reactivity (nicotine vs. neutral)	ROI (PCC) *control seeds: mPFC, anterior insula*	Activity	= PCC cue-reactivity		*P < .05* corrected with permutation testing		*Pos. Cor.* ΔCigs/day & ΔPCC, PCC, mPFC *N.S. Cor.* ΔCigs/day & anterior insula
Kragel, Sweitzer (30)		= Mindfulness (FFMQ), stress (PSS)	Cue reactivity (food vs. neutral)	Whole brain	Activity	↓ paraACC/SFG, fusiform, dmPFC, occipital, post-central/superior parietal, precentral, premotor		P < .05 cluster correction	Z = 4.09–5.93	
Kober, Brewer (29)^*^	↑ Craving post stress scenarios	↑ stress (VAS) post stress scenarios	Cue reactivity (stress vs. neutral)	Whole brain	Activity			*P < .05 FWE-corrected*		
Tang, Tang (9)	↓ Craving, CO		Rest (eyes closed)	Whole brain (fALFF)	Activity	↑ ACC/mPFC & IFG/vlPFC		*P < .05* montecarlo -corrected	*t = 3.66–4.99*	* N.S. Cor.* Craving, CO & ACC/mPFC, IFG/vlPFC

*Cross−sectionally, post intervention; ↑ Increased; ↓ Decreased; Δ Change; *=* no change

Abbreviations: ACC, anterior cingulate cortex; aDMN, anterior default mode network; CO, carbon monoxide; Cor, correlation; dmPFC, dorsomedial prefrontal cortex; DTS, distress tolerance scales; fALFF, fractional amplitude of low−frequency fluctuations; FFMQ, five facet mindfulness questionnaire; FMI, Freiburg mindfulness inventory; ICA, independent component analysis; IFG, inferior frontal gyrus; MBI, mindfulness−based intervention; mPFC, medial prefrontal cortex; N.S., non−significant; Neg, negative; PCC, posterior cingulate cortex; pDMN, posterior default mode network; Pos, positive; PSS, perceived stress scale; ROI, region of interest; rSFG, right superior frontal gyrus; SFG, superior frontal gyrus; UPPS−P, impulsive behavior scale; VAS, visual analogue scale; vlPFC, ventrolateral prefrontal cortex

Finally, we summarized results about the associations between MBI and behavior (e.g., substance use, mindfulness, and their changes), brain function, and the relevant brain-behavior associations across all studies. All neurobehavioral results were first summarized across studies and then as a function of the specific design used to aid interpretation in relation to: (a) *group-by-time* effects (Table 2), (b) MBIs vs. control groups at post-intervention (Table 2), (c) change *pre-to-post within MBIs* (Table 3), and (d) change *pre-to-post within control* interventions (Supplementary Table 10). The separation of the neurobehavioral results in distinct tables was undertaken to delineate the treatment effects from the effects for time within groups and group effects. Summaries of data were comprised of counts of the number of papers that reported specific features of the data extracted. Data from individual studies was summarized in tables and synthesized in narrative summaries.

### Data synthesis

All results were summarized by counting the number of studies which reported a specific feature in relation to the variable extracted.

## Results

Table 1 shows the characteristics of the 7 studies included [9, 19, 26–30]. All studies were published within the last 10 years—between 2013 and 2019—and examined tobacco users with one exception, which examined abstinent opioid users [26]. As shown in Table 1, individual studies that compared MBI and control groups had no significant differences in ages, and male-to-female proportions, with the exception of two studies that recruited a MBI group only [19, 30].

### Sample socio-demographic characteristics

The samples comprised 245 participants (of which 105 were female), with a mean age of 40 (range: 18 to 50 years), although one study reported minimum age instead of mean age [9]. Further details of the sociodemographic characteristics, substance use and misuse measures, and participants motivation to quit can be found in *Supplementary Text 1.*

### Overview of the interventions

MBI interventions varied and included general mindfulness training for smoking cessation (2 studies) [28, 29], and general mindfulness/mindful eating training [30] followed by the other interventions examined by individual studies: Mindfulness Orientated Recovery Enhancement (MORE) [27, 31], Mindfulness-based Therapy (MBT) [26], Integrative Mindfulness Body Training (IMBT) [9] and a Mindful Attention task [19] performed in the MRI scanner. All MBIs were manualized except for Mindfulness Training, which varied between studies.

Control interventions were used in all but 2 studies and varied to include passive control in one study (i.e., time-control comparison), and active control conditions: treatment as usual (TAU), National Cancer Institute’s QuitGuide app, Freedom from Smoking (FFS) and relaxation therapy.

Further details on the intervention type can be found in *Supplementary Textbox 1*, and the intervention parameters (mode of delivery, duration, and frequency) can be found in *Supplementary Text 2* and Supplementary Table 8.

### Summary of study designs and randomization procedures

The designs of the studies are overviewed in Supplementary Tables 8, and the time of administration of the interventions or fMRI testing or both is shown in Supplementary Table 9. The designs used to measure brain function in relation to MBI varied across studies. Three studies examined brain functional changes over time in MBI vs. control intervention groups and reported *group-by-time interactions* [9, 26–28]. Four studies [9, 26, 28, 30] measured brain functional changes *pre-to-post MBI*, and three of these studies also measured brain functional changes *pre-to-post in control interventions*. Two studies entailed a cross sectional comparison of brain function *at the conclusion of MBI and the control intervention* [9, 29]. One study only measured brain function *following a single MBI*, by comparing the of passive viewing of images with viewing images while applying mindfulness strategies [19].

Study designs were: two randomized controlled trials (RCTs) [28, 29], one randomized controlled experiment [9], one randomly assigned to any of the two groups (MBI, control) [26], one non-RCT [27] and two within-subject designs (i.e. MBI assessed at baseline and follow-up without a control group [30] and a within-subject control condition design where the same participant’s brain function during cue reactivity was measured and compared during a mindful state and a neutral state [19]. Details pertaining to the allocation of condition for participants can be found in *Supplementary Text 1.*

### MBI-associated changes in substance quantity and craving

#### Changes in drug quantity (number of cigarettes per day)

The single study on opioids did not report frequency of use at any time-point and participants were abstinent at baseline [26]. The two studies on smokers that reported changes found time-by- group interactions, with decreases in cigarette consumption in the MBI condition (*d =* 2.06; Tables 2 and 3) [27, 29]. In one RCT, there was an effect of time on decreased cigarette use *pre-to-post* MBI condition (*d* = 2.05; Table 3) and *pre-to-post control* condition (*d =* 1.28; Supplementary Table 10), but there was no *group-by-time* interaction effect (Tables 2; 28).

#### Changes in carbon monoxide levels

The two studies that examined levels of carbon monoxide levels reported intervention-by-time interactions [9, 27]. Carbon monoxide decreased in the MBI condition but not in the active control (*d* = 0.791) [27] and active relaxation condition (reduction = 60%; Tables 2 and 3, Supplementary Table 10) [9].

#### Changes in craving

Four studies examined changes in craving [9, 19, 27, 29]. Decreased cravings emerged *post MBI vs. control intervention* (VAS scores; *η*^*2*^ = 0.36) [19] and *pre-to-post MBI* (Table 3) but in the latter study there was no significant *group-by-time* effect (Table 2) [9, 27]. Cravings did not change *pre-to-post control* intervention (Supplementary Table 10) or during stressful vs. neutral scenarios during an emotional processing fMRI task [29]. A single study reported no change in **urges** to use substances during MBI, compared to a *control intervention* (Tables 2; 27).

### MBI-associated changes of mindfulness levels

The two studies [26, 30] measured mindfulness levels that reported mixed findings: there were effects for time with no statistical *group-by-time* interactions—whereby in the Freiburg Mindfulness Inventory (FMI) [26], scores significantly increased both *pre-to-post MBI* and *pre-to-post control* TAU (Table 3; Supplementary Table 10) in addition there were non-significant change *pre-to-post in the MBI condition* (Table 3) [30].

### Other MBI-associated changes in other behavioral measures (stress, affect, impulsivity)

#### Change in self-reported stress

Mixed results (decreases, increases, no change) emerged from the four studies that examined changes in stress via Visual-Analogue Scales (VAS), Distress Tolerance Scale (DTS) and Perceived Stress Scale (PSS) [19, 26, 29, 30]. Stress scores were reported to decrease when people were mindfully viewing cigarette-related pictures vs. when passively viewing [19]. Stress scores were also reported to significantly increase *pre-to-post* both the MBI and the control intervention (stress tolerance/regulation DTS scores), and *pre-to-post MBI* only (stress appraisal/absorption, Table 3; Supplementary Table 10) [26].

Non-significant differences in stress levels were reported post MBI vs. control (Freedom from Smoking) [29]; nor were differences found *pre-to-post for MBI integrative body-mind training* [9].

#### Changes in affect and impulsivity

A single study [27] reported a significant group by time interaction for positive affect with an increase over time for the *MBI condition but not for the control intervention (d* = 2.02*).* However, there were no changes in either group in either negative affect (PANAS) or VAS affective ratings (Table 2). Another study reported significant decreases in specific dimensions of impulsivity (i.e. negative affect from the UPPS-P) *pre-to-post MBI* (Table 3) and *pre-to-post control* intervention (e.g., sensation seeking, Supplementary Table 10) [26].

### Overview of fMRI methodologies

This section summaries the fMRI tasks, metrics and analyses used to examine intervention effects including intervention-by-time effects (Table 2), pre-to-post MBI effects (Table 3) and pre-to-post control intervention effects (Supplementary Table 10).

#### fMRI tasks

Brain function was measured mostly via cue reactivity fMRI tasks whereby people watched images of substances (e.g., cigarettes [28] [27, 32]) or natural rewards (e.g., food [30]) and of neutral stimuli (e.g., pens; n = 4 studies [28] [27, 29, 32]). Two single studies measured brain function using emotional processing fMRI tasks in which people viewed images and/or scripts about emotions: a stress reactivity fMRI task [29] and a passive emotion viewing fMRI task [27]. Two studies also measured brain function during rest (in addition to other fMRI tasks), while participants were instructed to close their eyes and let their mind wander [9, 26].

#### Overview of fMRI-outcome metrics and analyses

The reviewed studies examined brain *functional activity* during various fMRI tasks (n = 5) [19, 27, 29, 30] and *functional connectivity* during rest and fMRI tasks (n = 4) [9, 19, 26, 27]. One study reported results on activity and connectivity on a subset of participants (47 out of 54; [19]).

*Brain activity* was measured using exploratory whole-brain analyses, with the exception of one study [28] which used a hypothesis driven region-of-interest (ROI) approach and focused on the activity of posterior cingulate cortex, and two control seeds (i.e. medial prefrontal cortex, anterior insula).

*Resting-state functional connectivity* during rest (n = 2) [9, 26], was measured via exploratory analyses: whole-brain Independent Component Analysis (ICA) that identifies temporally coherent functional networks [26]; and amplitude of low-frequency fluctuation (ALFF) that quantifies “spontaneous” neural activity [9].

*Task-based functional connectivity* was measured using seed-to-whole brain connectivity [19, 27], whereby the activity of a priori, hypothesis-driven regions (termed ‘seeds’) is correlated with that of all other regions of the brain. The regions used as seeds were determined by the location of the brain activation during relevant emotion and stress fMRI task contrasts [27] and the subgenual cingulate cortex (sgACC) during a cue reactivity fMRI task (cigarette vs. neutral stimuli) [19].

### fMRI results: MBI-associated changes in brain function in substance use disorders

We aimed to identify the most consistently reported location of brain changes in the emerging literature. Therefore, findings summarized in this section refer to brain function (i.e., activity and connectivity).

All studies reported significant changes in brain function associated with MBI, most consistently within the ACC (*n* = 4) [9, 19, 27, 30] and the striatum (*n* = 3) [19, 27, 29], followed by other regions (n = 2): insula [19, 29], cerebellum [9, 29], precuneus [9, 19], inferior frontal gyrus [9, 26], and PCC [9, 28].

*Group-by-time* interactions were examined in three studies (Table 2) [26–28]. They showed decreases in the function of the inferior frontal gyrus (IFG) during rest, and both decreases and increases in the activity of the ACC and the striatum activity during two fMRI tasks (emotion processing/cue reactivity) with no effect on the PCC [28].

The group comparisons *after completion of the MBI vs. control intervention* (Table 2) in two studies indicated altered function in partially overlapping regions: the striatum (n = 2, e.g., ventral striatum, caudate, putamen) and other areas (e.g., cerebellum, precuneus, and insula). Single studies reported post- MBI vs. control intervention differences in the ACC and other frontal, subcortical, and visual regions (e.g., PCC, amygdala, fusiform gyrus).

Four studies compared brain function *pre-to-post MBI* (Table 3). Of these, two reported changes within the ACC and the SGF [9, 26, 30], and single studies reported both changes in other cortical regions (e.g., dmPFC, fusiform, occipital, postcentral/sup, precentral, premotor,IFG/vlPFC) and no change in the PCC [28].

Of the three studies that measured brain functional changes *pre-to-post control* interventions (Supplementary Table 10), only one reported significant changes in striatal (pallidum), temporal (temporal gyrus, hippocampus) thalamic and precentral intervention [26].

### Associations between changes in brain function and behavioral measures

#### Brain functional changes and daily cigarette use

Four studies examined associations between brain function and cigarettes per day/magnitude of smoking reduction and reported mostly positive and significant correlations [9, 27–29].

In studies that reported a group by time effect on brain function, positive correlations were reported between degree of reduction of cigarette use and degree of increase in activity of the rACC (*r* = .91) and ventral striatum (*r* = .68) (Table 2) [27]. A separate study did not report a significant *group-by-time* effect on brain function, but it reported significant positive correlations between decrease in number of cigarettes/day *pre-to-post MBI* decrease in activity of the PCC (*r* = .39) and medial PFC (*r* = .35) *pre-to-post MBI* (Table 2) [28]. A third study reported significantly greater brain activity *post MBI* vs. *post control* intervention [29]. The investigators found significant positive correlations between activity of the medial cortico-temporal regions (anterior insula, amygdala and para-hippocampus) and the number of daily cigarettes measured at baseline (*d* = 1.18) [29].

No study conducted correlations between decreases in cigarette use and brain activity changes *pre-to-post control* interventions (e.g., National Cancer Institute’s QuitGuide, treatment as usual; Supplementary Table 10) [26, 28, 29]; or between carbon monoxide and any brain functional measure [9].

#### Brain functional change and mindfulness levels

One study that reported a significant group by time effect on brain function, reported a correlation between the change in brain function associated with MBI and treatment related change *over time* in mindfulness levels measured with FMI scores [26]. Increased mindfulness levels *pre-to-post MBI* were associated with decreases in IFG connectivity *pre-to-post MBI* (*r* = -.464; Table 2), and with *pre-to-post MBI* decreases in SFG connectivity (*r* = -.594; Table 3). There was no correlation between changes *pre-to-post control intervention* in mindfulness levels and those in DMN connectivity (Supplementary Table 10).

#### Brain function and other psychological measures

Individual studies reported brain-behavior correlations, between: (i) brain functional changes that emerged from *intervention-by-group*, *pre-to-post MBI* and *pre-to-post control* interventions (Tables 2 and 3; Supplementary Table 10); and (ii) measures of (baseline or changes in) craving, stress (tolerance, appraisal, absorption, regulation, DTS), positive and negative affect (PANAS), and different dimensions of impulsivity (UPPS-P) [26, 27].

For the studies that reported *group-by-time* effects, significant brain-behavior correlations emerged. There were negative correlations between increased activity of the ventral striatum associated with MBI, and *decreases* in urges to smoke *pre-to-post MBI* (*r* = -.7) [27]. Positive correlations were reported between changes *pre-to-post MBI*, specifically increased rACC activity (*r* = .614) and rACC-OFC connectivity (*r* = .635) with increased positive affect (PANAS) [27].

## Discussion

The emerging evidence from 7 studies indicates that brain functional changes associated with MBI may occur in individuals diagnosed with SUDs in pathways relevant for reward processing [4] and for mindfulness, most consistently the ACC and the striatum [10, 33, 34]. Brain function was largely unaffected by control interventions. The reviewed MBIs reduced quantity of cigarette use and cravings, and brain functional changes were associated with lower quantity of use. The evidence is insufficient to confirm whether MBIs in the reviewed studies changed mindfulness states or traits or both, or mental health symptom scores. Further, there is little-to-no evidence about how MBI affects psychological wellbeing in SUD, or how brain functional changes in individuals diagnosed with SUD correlates with measures of mental health symptom severity.

The evidence to date shows that MBIs are associated with brain functional changes (activity and connectivity), particularly within the ACC and the striatum. Notably, these regions are implicated in neuroscientific theories of addiction [4] and meta-analytic evidence on the effects of mindfulness training [10, 35]. The ACC is implicated in addiction-relevant cravings, motivation and preoccupation with substance use [4]; and also is targeted by mindfulness to aid self-regulation and conflict-monitoring [4, 10, 36].

The striatum is a core component of reward processing, sensitivity to reward and compulsive substance use [35]; and meta-analytic evidence from healthy samples indicates that it is engaged by meditative states [10]. Thus, MBIs may regulate the function of key cortical/inhibitory regions within the mindfulness network that are aberrant in addiction (e.g., ACC). This might occur by MBIs mitigating cortical dysfunction (e.g., within the ACC). Specifically, MBIs may boost cortical inhibitory projections that downregulate reactivity of striatal regions implicated in craving – thereby changing the connectivity between these regions or the activity of the regions, or both. Indeed, MBIs were consistently associated with lower craving and drug quantity consumption across the behavioral literature to date [18] and in the current review of fMRI studies.

The reviewed nascent literature showed that MBIs were also associated with changes (mostly with small-effect sizes) in the function of additional and interconnected regions implicated in cognitive processes that are core to both reward dysregulation and to mindfulness. They include: the insula, cerebellum, precuneus, inferior frontal gyrus, and PCC. MBIs in the examined SUDs were associated with changes in the function of the insula, and some of these effects were reported to be robust (e.g., whole-brain family-wise corrected) [29]. The insula is a primary hub for interoception (i.e., awareness of bodily states, which in addiction could be affected by cravings, withdrawal). Notably, altered interoception and cravings in addiction [37–39], as well as relapse [40], have been ascribed to the insula. Indeed, the insula has been a target of brain stimulation to diminish appetitive behavior [41, 42] and its function during inhibition to smoking cues has been correlated with abstinence (i.e. to tobacco) [43]. Concurrently, the insula has been recognized as one of the primary neural mechanisms of action for mindfulness practice in healthy samples [44]. For example, mindfulness practice has been shown to down-regulate the activity of the insula (voxel-wise *P* < .01, exceeding volume threshold of 256 µl for cluster wise probability of 0.05 for the insula), as tested during an inspiratory breathing fMRI task in a sample of marines before and after undergoing 20 h of mindfulness-based fitness training or training as usual [36]. Also, mindfulness practice has been associated with medium-effect size changes of the structural connectivity of the insula network, compared to a control cognitive training intervention [45]. Overall, early evidence that MBIs in SUD may involve modulation of the insula is consistent with existing emerging findings from addiction and mindfulness literature. Interestingly, while insular function decreased pre-to-post MBI in the examined SUDs, in healthy samples findings pertaining to MBI-associated changes in insular function have been mixed. For example, in a recent systematic review and meta-analysis on mindfulness fMRI tasks in healthy samples, only 62% of fMRI studies using MBI reported increased activity of the insula [46]. The direction of changes in insular function in the examined SUDs and healthy samples may be (partly) due to distinct baseline levels of insular function, which are aberrant in SUD compared to controls and may be more heterogeneous in healthy samples.

The emerging evidence synthesized in our review revealed SUD-related changes in additional higher order cognitive control regions (precuneus, IFG). Importantly, these regions are reportedly implicated in executive dysfunction in SUDs (e.g., disinhibition, self-control, salience attribution and awareness, compulsive use) [33]. In addition, they have been implicated in substance use behavior (e.g., smoking and drinking quantities) [47]. The inferior frontal gyrus has been implicated in inhibitory control during withdrawal (i.e., from smoking) [48], disinhibition [49], intoxication [50], and in relapse [40]; while the precuneus is implicated in reactivity to drug cues (i.e., tobacco and alcohol; [51]) and impulsivity [52]. In addition, mindfulness practice has been shown to modulate higher cognitive control regions [53, 54]. Together, the findings provide support for the hypothesis that MBIs affect inferior frontal/precuneus regions implicated in addiction.

The reviewed evidence also showed that MBIs change the function of the PCC [9, 28]. Notably, the PCC underlies the cognitive processes of self-reference [55] and is a core component of the default mode network, a brain system that activates ‘when individuals are not focused on the external environment’ [56, 57]. Previous work shows that the function of the PCC is altered in addiction, including in the population diagnosed with SUDs as examined in this review (i.e., nicotine, opiate disorders, alcohol use disorders) [58] and while performing fMRI tasks used in most of the reviewed studies (i.e., resting state and cue reactivity) [59, 60]. The emerging findings from the literature are aligned with theories that meditation changes the function of the PCC [11], and with growing evidence that MBIs change the function of the PCC in healthy samples (e.g., connectivity) [12–14].

Further, the evidence from this review indicates that changes in PCC function correlates with cigarette reduction. This finding is consistent with previous work that structural damage of the PCC disrupts cigarette smoking [61] and functional connectivity predicts relapse [62]. Future work is required to confirm that the PCC is implicated in reduced use of other substances and to explore its role in predicting relapse post MBIs.

Changes in the function of the cerebellum also emerged in fMRI studies of mindfulness in the examined SUDs, consistent with findings that it is implicated in meditative states [10]. These findings are also in line with evidence that the cerebellum is an integral part of the addiction circuitry [63] and plays a key role in reward processing, motivation and cognitive control.

**Fig. 1:**
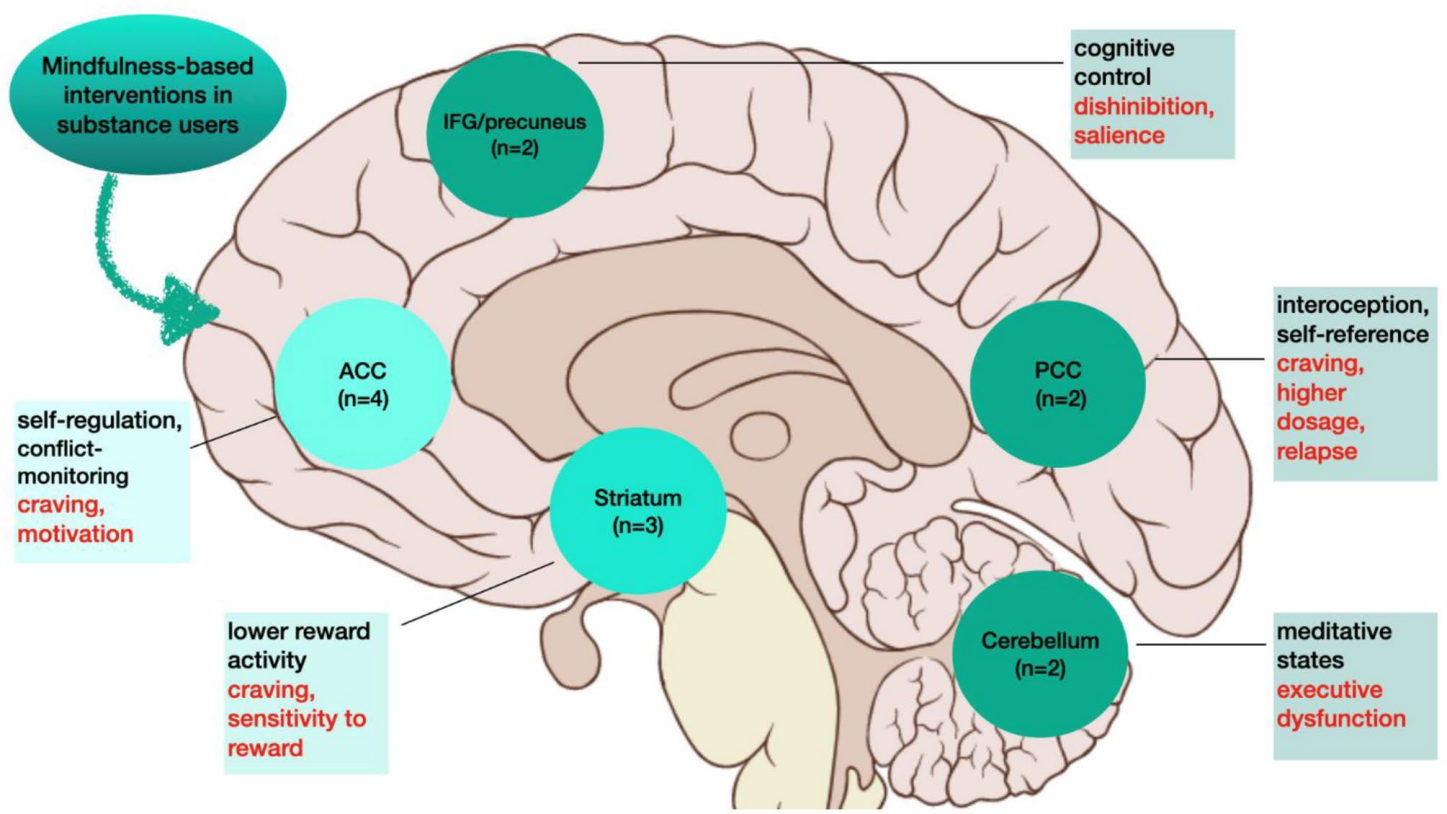
Visualization of emerging pattern of brain functional changes with mindfulness-based interventions in substance use disorders from the functional neuroimaging literature to date. ACC = anterior cingulate cortex; IFG = inferior frontal gyrus; PCC = posterior cingulate cortex. Dark green = 4 studies (ACC); moderate green = 3 studies (striatum); light green = 2 studies (IFG, precuneus, PCC and cerebellum). In textboxes, black fonts indicate cognitive functions each area is implicated in; and red fonts refer to addiction-relevant processes each area has been ascribed to

In sum, the reviewed evidence suggests that brain functional changes are associated with MBIs in the examined SUDs (i.e., opiates, nicotine; see Fig. 1), most consistently the ACC, followed by the striatum, and other regions (e.g., insula, precuneus/inferior frontal gyrus, PCC, cerebellum). This network of regions has previously been implicated in SUDs [33, 64], MBIs and mindfulness practice in healthy samples and cognitive-based interventions in SUDs [65]. Therefore, it may be that MBIs target the function of core regions of the addiction neurocircuitry.

Changes in the ACC were reported also by Fahmy and colleagues, who conducted the only study in opiate users, who were in treatment (with mood stabilizers, antipsychotics and sedatives) [26]. Thus, MBIs may ameliorate dysregulated reward function of the ACC across the examined SUDs, and in response to natural and drug related rewards. At this point there is insufficient evidence to conclude that MBIs affect distinct brain pathways that might be relevant to specific SUD populations, which is worthy of investigation given that there are important differences in the psychopharmacological signatures of specific drugs. Future well-controlled intervention studies that deliver consistent MBIs to distinct SUD groups are required to unpack this.

Several studies reported correlations between brain functional changes associated with MBI (i.e., striatum, ACC, PCC, mPFC) and reduction in cigarettes exposure [9, 27–29]. Thus, brain functional changes associated with MBI may underlie reduction in substance use quantity as found in this review (i.e., decreased cigarette use) [27, 29], and previous meta-analyses (i.e., several substances) [18]. However, as the nature of the association is correlational the opposite may be true – reduction in quantity of substance use may drive changes in brain function.

Of note, variables associated with changes in drug quantity were not systematically measured or accounted for (e.g., withdrawal, craving, nicotine receptors), and their role remains to be clarified. Further in some studies, changes *pre-to-post MBIs* were noted in brain function but not in problems with substance use (e.g., craving). This trend may reflect a dissociation between neurocognitive and behavioral changes in MBI – whereby changes may occur in brain function but not in behavior—as previously suggested [66]. Alternatively, there could be a threshold of change required for brain related change to drive behavioral change. To test this hypothesis, growth models that allow for random intercepts and slopes are warranted. A strength of the literature was that all sample characteristics (e.g., demographics, mindfulness, personality measures, craving, substance exposure) were reportedly not significantly different between intervention groups at baseline. Therefore, the group differences in brain function are unlikely to reflect characteristics predating MBIs. However, non- random assignment of participants to groups in some studies, and other potential biases need to be accounted for when drawing conclusions about the strength of the evidence.

Interestingly, trait mindfulness has been seldom examined in the reviewed literature and in relation to brain function. In one study conducted by Kober and colleagues (2019), non-significant trends emerged for an intervention-by-time effect on trait mindfulness (FFMQ [67]. Specifically, participants in the MT group (but not those in the FFS group) showed increased scores on trait mindfulness facets including “observe”, “non-react” and total score. Meanwhile, the MT group (vs FFMQ and CBT) showed lower stress reactivity of the amygdala and insula, which was associated with reduced smoking post intervention and at 3-month follow-up. Thus, it cannot be excluded that changes in trait mindfulness specific to the MT group might have moderated reduced stress brain reactivity. In line with this notion, greater trait mindfulness has been associated with reduced self-reported distress and negative affect in response to laboratory stressors [68]; and amygdala’ reactivity to negative faces [69] and amygdala function during rest [70]. Given that only a single fMRI study has examined trait mindfulness in relation to MBI related brain functional changes in the examined SUDs, more evidence is required to demonstrate if trait mindfulness moderates how MBIs affect brain function in SUD.

There is insufficient evidence to demonstrate if brain functional changes associated with MBIs cause changes in mindfulness. Only one study that reported a *group-by-time* effect on brain function, explored and demonstrated correlations between mindfulness changes *pre-to-post MBI* and brain functional changes in higher order cognitive control regions (IFG) [26]. Further, there were limited and mixed findings regarding how MBIs affected mindfulness levels in the reviewed studies, with lack of changes and increases in mindfulness levels in both MBI and TAU [26, 30]. The evidence synthesized in this review contrasted meta-analytic findings of increased mindfulness scores with MBIs in people diagnosed with a SUD [18]. The lack of robust change in mindfulness in the reviewed samples may be due to methodological limitations of the fMRI studies in SUDs. First, only two studies examined changes in mindfulness scores [26, 30] and their sample sizes were inadequately powered to robustly detect changes (n = 5 and 16 participants per group). Thus, there is insufficient evidence to determine if the reviewed interventions changed mindfulness levels.

Second, mindfulness was measured via two distinct scales that did not assess mindfulness in relation to the period of time that synchronized with the intervention (e.g., past week, 3 weeks) or did not specify an index period of time or both; therefore, they might have not been sensitive to any changes over the specific intervention period. Changes in mindfulness may have been evident if appropriate measures had been used immediately before and after each mindfulness session, such as the State Mindfulness Scale, which has been shown to be sensitive to mindfulness changes in healthy samples [71].

Overall, there is insufficient evidence to determine if MBIs in the reviewed studies increased mindfulness, and if brain changes in SUDs associated with MBIs correlate with or are caused by increased mindfulness. These hypotheses require testing through future repeated measure fMRI studies with active and passive control conditions, that assess dimensions of mindfulness relevant for the intervention type and duration, to detect mindfulness changes with precision. Similarly, the evidence on brain functional changes associated with MBI and urges, stress, dimensions of personality, mental health and wellbeing consisted mostly of single studies. The fMRI evidence to date is insufficient to profile the relevance of brain functional changes of MBIs in addiction and warrants detailed assessment of measures of mental health and wellbeing and their relationship to brain function.

### Overview of limitations and direction for future work

The findings of the reviewed literature need to be interpreted considering methodological limitations.

First, only a few relevant studies have been conducted thus far, and these have used relatively small sample sizes, with two studies including less than 20 participants (range: 5 to 67; median: 32). Despite emerging trends summarized herein, investigations have not been consistently powered to reliably detect significant treatment effects on brain function. The findings to date require confirmation by replication studies with a priori power analyses to estimate the required sample sizes. Indeed, the effect sizes of the brain changes were small and may remain undetected in future studies without adequate statistical power. Future work should also systematically report the effect sizes in relation to the main neurobehavioral outcome variables.

Second, the designs of the reviewed studies were heterogeneous in relation to: (i) active and passive control interventions, which precluded the systematic assessment of the emerging effects of MBIs on the brain; (ii) within-group, between-group and within-between group designs, meaning that not all findings accounted for baseline group differences in neurobehavioral data that could have confounded the intervention-related results; and (iii) treatment allocation, randomization and concealment procedures, which might have introduced risks of bias (e.g., expectancy effects). Confirmatory work to robustly isolate neurobiological changes associated with MBIs is warranted using within-between designs to detect the effect of MBIs on brain function, accounting for baseline differences and the control intervention; and both active placebo control comparison (i.e., to account for the effects of an active treatment) and passive placebo control comparisons (i.e., to parcel out the effect of time without treatment).

Third, there are limits to the generalizability of the findings pertaining to brain functional changes in relations to: substance use disorders with high prevalence across distinct world regions, across MBIs other than the ones reviewed herein (e.g., mindfulness-based relapse prevention [MBRP]) and the brain systems examined (e.g., underlying resting state and cue reactivity). Indeed, all samples were comprised of samples recruited in the USA, cigarette users (one sample was opioid users), and brain function was limited to the brain systems engaged by cue reactivity and resting state fMRI tasks. On the other hand, a strength of the reviewed literature is that the MBIs used (e.g., MORE, MBT, IBMT) are evidence based [31, 72–75].

Replication fMRI studies on samples recruited from distinct countries where SUDs are represented are required. They are required to confirm the neurobiological mechanisms of MBIs in samples with cigarette and opiate use disorders, and other highly prevalent SUDs (e.g., alcohol, cannabis, cocaine use disorders, gambling disorders), and to measure brain activity and connectivity using other fMRI tasks. Similarly, more studies using the MBIs reviewed herein and other effective MBIs (e.g., MBRP) [18] are required to identify shared and distinct effects of distinct MBIs on neurobiology, across distinct types of MBIs. In particular, confirmatory work is required with the most effective and manualized MBI approaches shown to be most effective in the treatment of SUDs and with defined psychological/cognitive mechanisms (e.g., MBRP).

Notably, MBI was the sole intervention in all studies of cigarette users, who were not undergoing other therapies. Thus, the brain changes in cigarette users are not attributable to other current therapies; even though it cannot be excluded that specific past therapies may have facilitated brain functional plasticity in response to MBI. MBI was used in conjunction with treatment-as-usual only in opiate users, therefore the effects on this group specifically are entrenched with those of existing treatments. Future work is required to determine how MBI alone and as an adjunct to interventions targeting SUDs affect the brain.

Fourth, measurement issues prevented the understanding of treatment related changes in behavior and brain function: (i) the behavioral and MRI data were often collected at inconsistent time points, with some studies collecting fMRI data at baseline and follow-up and behavioral data only at baseline—therefore it was not possible to consistently assess how brain functional changes associated with MBI paralleled those in behavior, or the sequence of these changes; (ii) limited behavioral data was collected (e.g., only 2 studies measured mindfulness), which limited the understanding of how MBIs affected behavior and clinical outcomes; (iii) some of the behavioral data was collected at baseline only (e.g., mindfulness levels), thus we could not examine if the MBIs targeted and increased mindfulness; (iv) some variables key to treatment (e.g., adherence, motivation to change, experiential avoidance—avoidance of cravings and associated discomfort) as a target of mindfulness in SUDs) were not measured, which prevents the understanding of the clinical significance of the findings. Specifically, this issue limits the capacity to conclude that mindfulness change occurred through the expected psychological mechanisms. Future work is required to measure concurrently neural and behavioral data that could be affected by MBIs, at all assessment times; and to measure in detail variables that can influence treatment response.

Fifth, the fMRI studies are heavily skewed towards cigarette users. This contrasts the clinical trial literature on MBIs, which relies on samples with SUDs of a variety of substances (e.g., nicotine, alcohol, and other substances, cocaine, cannabis, methamphetamine), and of addictive behaviors (e.g., problem gambling, binge eating) [15, 18]. The focus on cigarette users may reflect problems with the retention of participants who use substances other than nicotine, and poly-substance users. Future studies are required to focus on strategies to recruit and retain more participants with distinct SUDs—multi-site studies that recruit participants across different locations may prove useful to this end.

Of note, dual diagnoses were excluded to minimize the impact that mental health disorders can have on neurobiology independently and in interaction with SUD. Given the high levels of comorbid anxiety and depression in SUD, the reviewed samples might be unrepresentative of the SUD population. Yet, it cannot be ruled out that some of the participants included in the sample had a dual diagnosis—or elevated subclinical symptoms—of mental health disorders –that affect brain function and are associated with SUD [76–78]. Presence/symptoms of mental health disorders were not systematically accounted for in the analyses, and therefore may have confounded the results.

Additional methodological limitations prevented the understanding of how MBI-associated brain functional changes in SUDs affect change in variables that could have been affected by the intervention e.g., changes in substance use/misuse, mental health, mindfulness/wellbeing as well as in cognitive measures targeted by the intervention. First, a handful of studies on relatively small sample sizes examined the correlation between brain functional changes and behavioral variables. Therefore, there is insufficient evidence to conclude that brain functional changes were strongly associated with behavioral measures, or that changes in mindfulness mediated the effect of MBIs on neurobehavioral outcomes. Second, some of the behavioral variables used in correlations were measured at a single time point and not *pre-to-post MBI*, and some correlations were run in the MBI and control groups. Therefore, some of the brain-behavior correlations did not reflect behavioral changes that occurred at a function of the intervention.

Future studies should confirm how MBI-associated brain changes drive changes reported in the literature to date, in substance use/misuse, mental health (including mindfulness levels) and wellbeing, via careful measurement of these variables at all assessment points, and correlation of outcomes within intervention groups separately. Additionally, changes in cognitive functions ascribed to mindfulness (e.g., focused attention, inhibitory control) should also be examined in relation to brain function in order to elucidate the relevant cognitive mechanism of change of the intervention.

Finally, there was an overall moderate risk of bias in the reviewed findings, which may be mitigated by future work which adheres to standardized guidelines for the risk of bias such as those described by Young, van der Velden (22). Specifically, more rigorous evidence is required by using RCTs, the use/description of randomization procedures and of intervention characteristics (e.g., attendance, teacher training required, manualized intervention); the measurement of baseline differences between intervention groups and the implementation of methodologies to account for any confounding effects (e.g., using a within-between subject design to account for baseline differences); and to conceal treatment allocation (e.g., using active and passive placebo control conditions). Additionally, the quality of reporting of fMRI methodologies was overall moderate-to-high with most studies providing detailed reporting. In order to improve existing standards, future work is warranted to report all required methodological variables (e.g., number of volumes) and of participants’ characteristics which may confound fMRI results (e.g., handedness). Therefore, we recommend the conduct of replication studies using high quality methodological standards and we provide recommendations for consideration for future work in Textbox 1.

#### Textbox 1. Recommended minimum methodological standards for future fMRI work of MBI in SUDs

1. Pre-registration of the study in order to minimise any biases.
2. *Use of robust designs* to disentangle MBI-related effects from those of time and treatment, e.g., RCTs, use of passive and active placebo-controlled conditions and of within-between subject designs, adequately powered samples to detect the effects of interest (e.g., a priori power analyses informed by growing evidence).
3. Adherence—from study conception—to quality standards for minimising risk of bias (e.g., Young and colleagues, 2018), and increasing quality of reporting for fMRI studies.
4. Recruitment of samples comprising women and men from diverse socio-economic backgrounds and distinct world regions, to aid the generalisability of the findings.
5. Concurrent measurement of fMRI and relevant behavioural data that could be affected by MBIs, at all assessment times.
6. Measure concurrently neural, substance use/misuse (e.g., craving, drug quantity), mental health (e.g., mood), psychological wellbeing and cognitive variables (e.g., disinhibition) at all assessment times with adequate sensitivity to detect changes over the specific intervention period, to enable the understanding of MBI-related changes.
7. Measure variables related to treatment compliance (e.g., motivation to change, dropout) at during and after the intervention, to enable the understanding of how brain functional changes can predict treatment response.
8. Conduct exploratory correlations between brain function and behavioural measures separately in distinct treatment groups, to create an evidence-based for the most relevant behavioural outcomes across distinct interventions and substance user groups.
9. Measurement of brain function by combining *consistent resting-state fMRI tasks* the data of which that can be acquired relatively easy, in order to gather evidence to systematically integrate how mindfulness-based interventions affect brain function without cognitive confounds; and *study-specific fMRI task*s to test hypotheses on brain function during cognitive tasks of interest.
10. Measurement of brain function by combining unbiased, exploratory whole brain approaches to gather and integrate new evidence on most robust activation patterns resting-state tasks the data of which that can be acquired relatively easy, in order to; to complement a-priori analyses that focus on specific hypothesised brain pathways.
11. Confirmatory evidence using distinct manualised mindfulness-based interventions shown to be most effective in the treatment of substance use disorders (e.g., MORE, MBRP); in order to identify brain changes common and specific to distinct types of interventions.
12. Replication studies across substance use disorders including behavioural addictions, to examine if mindfulness-brain interventions are most effective at aiding brain plasticity in specific substance use disorders are brain changes common and specific to distinct types of interventions.

## Conclusions

This systematic review provides useful insights into functional brain changes associated with MBI in people with a SUD. The synthesis of findings revealed that MBIs improve brain function in chronic substance users in regions ascribed to substance-related stress, drug quantity, and cravings, specifically in the ACC and mPFC pathways, with small effect sizes. There was also preliminary evidence suggesting that MBIs-related improvement in behavioral measures of stress, mindfulness, and impulsiveness, was associated with brain functional changes in the insula, ventral striatum, and IFG. Given the low number of behavioral measures used, it is premature to draw any conclusions about MBI-associated brain changes.

Therefore, these findings underscore the need for greater consistency in the use of robust methodologies (e.g., adequately powered analyses with larger sample sizes, correlations with behavioral measures, repeated measures) to provide high-quality and conclusive evidence on the neurobiology of MBIs in chronic substance users and other behavioral outcomes. Further neuroimaging research may improve the understanding of how MBIs functionally change the brain regions and networks implicated in chronic substance use, thereby leading to refinements that increases their effectiveness in treating these highly vulnerable populations.

## Electronic supplementary material

Below is the link to the electronic supplementary material.

Supplementary Material 1 Overview of methodologies for the searches, additional samples/studies characteristics and risk of bias assessment

## Data Availability

All data generated or analysed during this study are included in this published article and its supplementary information files.
