# Supplementary material for: Do mindfulness-based interventions change brain function in people with substance dependence? A systematic review of the fMRI evidence
Source: BMC Psychiatry. 2023 Jun 7;23:407. doi: 10.1186/s12888-023-04789-7 (PMC10246321; doi:10.1186/s12888-023-04789-7)
Supplement: Supplementary file 1 — Supplementary Material 1 Overview of methodologies for the searches, additional samples/studies characteristics and risk of bias assessment [file 12888_2023_4789_MOESM1_ESM.docx]

**Supplementary Materials**

Supplementary Table 1.

*PRISMA 2020 Checklist*

| **Section and Topic** | **Item #** | **Checklist item** | **Location where item is reported** |
| --- | --- | --- | --- |
| **TITLE** | | |  |
| Title | 1 | Identify the report as a systematic review. | 1 |
| **ABSTRACT** | | |  |
| Abstract | 2 | See the PRISMA 2020 for Abstracts checklist. | 1 |
| **INTRODUCTION** | | |  |
| Rationale | 3 | Describe the rationale for the review in the context of existing knowledge. | 2 |
| Objectives | 4 | Provide an explicit statement of the objective(s) or question(s) the review addresses. | 2-3 |
| **METHODS** | | |  |
| Eligibility criteria | 5 | Specify the inclusion and exclusion criteria for the review and how studies were grouped for the syntheses. | 3 |
| Information sources | 6 | Specify all databases, registers, websites, organisations, reference lists and other sources searched or consulted to identify studies. Specify the date when each source was last searched or consulted. | 3 |
| Search strategy | 7 | Present the full search strategies for all databases, registers and websites, including any filters and limits used. | Supplementary Material (3-8) |
| Selection process | 8 | Specify the methods used to decide whether a study met the inclusion criteria of the review, including how many reviewers screened each record and each report retrieved, whether they worked independently, and if applicable, details of automation tools used in the process. | 3 |
| Data collection process | 9 | Specify the methods used to collect data from reports, including how many reviewers collected data from each report, whether they worked independently, any processes for obtaining or confirming data from study investigators, and if applicable, details of automation tools used in the process. | 3-4 |
| Data items | 10a | List and define all outcomes for which data were sought. Specify whether all results that were compatible with each outcome domain in each study were sought (e.g., for all measures, time points, analyses), and if not, the methods used to decide which results to collect. | 3-4 |
|  | 10b | List and define all other variables for which data were sought (e.g., participant and intervention characteristics, funding sources). Describe any assumptions made about any missing or unclear information. | 3-4 |
| Study risk of bias assessment | 11 | Specify the methods used to assess risk of bias in the included studies, including details of the tool(s) used, how many reviewers assessed each study and whether they worked independently, and if applicable, details of automation tools used in the process. | 3; Supplementary Material (21-25) |
| Effect measures | 12 | Specify for each outcome the effect measure(s) (e.g., risk ratio, mean difference) used in the synthesis or presentation of results . | 3-4 |
| Synthesis methods | 13a | Describe the processes used to decide which studies were eligible for each synthesis (e.g., tabulating the study intervention characteristics and comparing against the planned groups for each synthesis (item #5)). | 3-4 |
|  | 13b | Describe any methods required to prepare the data for presentation or synthesis, such as handling of missing summary statistics, or data conversions. | 3-4 |
|  | 13c | Describe any methods used to tabulate or visually display results of individual studies and syntheses. | 3-4 |
|  | 13d | Describe any methods used to synthesise results and provide a rationale for the choice(s). If meta-analysis was performed, describe the model(s), method(s) to identify the presence and extent of statistical heterogeneity, and software package(s) used. | NA |
|  | 13e | Describe any methods used to explore possible causes of heterogeneity among study results (e.g., subgroup analysis, meta-regression). | 3-4 |
|  | 13f | Describe any sensitivity analyses conducted to assess robustness of the synthesised results. | 3-4 |
| Reporting bias assessment | 14 | Describe any methods used to assess risk of bias due to missing results in a synthesis (arising from reporting biases). | 3; Supplementary Material (21-25) |
| Certainty assessment | 15 | Describe any methods used to assess certainty (or confidence) in the body of evidence for an outcome. | 3-4 |
| **RESULTS** | | |  |
| Study selection | 16a | Describe the results of the search and selection process, from the number of records identified in the search to the number of studies included in the review, ideally using a flow diagram. | 4; Supplementary Material (9) |
|  | 16b | Cite studies that might appear to meet the inclusion criteria, but which were excluded, and explain why they were excluded. | N/A |
| Study characteristics | 17 | Cite each included study and present its characteristics. | 4-10 |
| Risk of bias in studies | 18 | Present assessments of risk of bias for each included study. | Supplementary Material (21-25) |
| Results of individual studies | 19 | For all outcomes, present, for each study: (a) summary statistics for each group (where appropriate) and (b) an effect estimate and its precision (e.g., confidence/credible interval), ideally using structured tables or plots. | 5-8 |
| Results of syntheses | 20a | For each synthesis, briefly summarise the characteristics and risk of bias among contributing studies. | Supplementary Material (21-25) |
|  | 20b | Present results of all statistical syntheses conducted. If meta-analysis was done, present for each the summary estimate and its precision (e.g., confidence/credible interval) and measures of statistical heterogeneity. If comparing groups, describe the direction of the effect. | N/A |
|  | 20c | Present results of all investigations of possible causes of heterogeneity among study results. | 8-10 |
|  | 20d | Present results of all sensitivity analyses conducted to assess the robustness of the synthesised results. | 8-10 |
| Reporting biases | 21 | Present assessments of risk of bias due to missing results (arising from reporting biases) for each synthesis assessed. | Supplementary Material (21-25) |
| Certainty of evidence | 22 | Present assessments of certainty (or confidence) in the body of evidence for each outcome assessed. | N/A |
| **DISCUSSION** | | |  |
| Discussion | 23a | Provide a general interpretation of the results in the context of other evidence. | 10-13 |
|  | 23b | Discuss any limitations of the evidence included in the review. | 13-14 |
|  | 23c | Discuss any limitations of the review processes used. | 14-15 |
|  | 23d | Discuss implications of the results for practice, policy, and future research. | 13-15 |
| **OTHER INFORMATION** | | |  |
| Registration and protocol | 24a | Provide registration information for the review, including register name and registration number, or state that the review was not registered. | 3 |
|  | 24b | Indicate where the review protocol can be accessed, or state that a protocol was not prepared. | 3 |
|  | 24c | Describe and explain any amendments to information provided at registration or in the protocol. | N/A |
| Support | 25 | Describe sources of financial or non-financial support for the review, and the role of the funders or sponsors in the review. | 16 |
| Competing interests | 26 | Declare any competing interests of review authors. | N/A |
| Availability of data, code and other materials | 27 | Report which of the following are publicly available and where they can be found: template data collection forms; data extracted from included studies; data used for all analyses; analytic code; any other materials used in the review. | NA |

*From:* Page MJ, McKenzie JE, Bossuyt PM, Boutron I, Hoffmann TC, Mulrow CD, et al. The PRISMA 2020 statement: an updated guideline for reporting systematic reviews. BMJ 2021;372:n71. doi: 10.1136/bmj.n71

For more information, visit: <http://www.prisma-statement.org/>

Supplementary Table 2.

*Search Strategy Across All Databases*

| ***PsycINFO*** *26/04/2021* | |  |
| --- | --- | --- |
| **#** | **Search Terms** | **Results** |
| **Concept 1: Substance** | | |
| **#1** | DE “Drug Dependency” OR DE “Drug Addiction” OR MM “Drug Usage” OR MM “Drug Abuse” OR MM “Drug Therapy” OR DE “Addiction Treatment” OR DE “Substance Use Disorder OR DE “Substance Use Prevention” OR DE “Substance Use Treatment” OR DE “Substance Related and Addictive Disorders” OR DE “Substance Abuse and Addiction Measures” OR DE “Polydrug Abuse” OR MM “Opioid Use Disorder” OR MM “Opiates” OR MM “Heroin OR MM “Alcohol Abuse” OR MM “Alcohol Treatment” OR MM “Alcohol Use Disorder” OR MM “Cocaine” OR MM “Amphetamine” OR MM “Cannabis” OR MM “Nicotine” | **228,345** |
| **#2** | drug* OR substance* OR narcotic* OR addict* OR heroin* OR opiate* OR opioid* OR opium OR alcohol* OR cocaine* OR “crack cocaine” OR methamphetamine* OR amphetamine* OR ice* OR ecstasy OR MDMA OR cannabis* OR marijuana* OR tobacco* OR nicotin* OR hallucinogen* OR ketamine OR LSD OR psilocybin OR DMT OR psychotropic OR polydrug* OR polysubstance* OR kratom OR prescription* OR “gamma hydroxybutyrate” OR GHB OR GBH; ti,ab | **658,102** |
| **#3** | #1 OR #2 | **658,102** |
| **Concept 2: Functional Neuroimaging** | | |
| **#4** | DE “Functional Magnetic Resonance Imaging” OR DE “Neuroimaging” OR DE “Brain Connectivity” | **48,973** |
| **#5** | fMRI OR functional MRI OR functional magnetic resonance imag* OR functional neuroimag* OR BOLD OR blood oxygen level-dependent OR brain function* OR brain connect* OR neural connect* OR functional connect* OR brain activity OR neural activity OR functional activity; ti,ab | **349,265** |
| **#6** | #4 OR #5 | **356,870** |
| **Concept 3: Mindfulness** | | |
| **#7** | DE “Mindfulness” OR DE “Mindfulness-Based Interventions” OR DE “Meditation” | **16,004** |
| **#8** | mindful* OR meditat* OR MBI OR MBSR OR MBCT OR MBRP; ti,ab | **28,627** |
| **#9** | #7 OR #8 | **28,627** |
| **#10** | #3 **AND** #6 **AND** #9 | **119** |
| - ***(MeSH) Subject Heading Command*** *signifies the specific controlled vocabulary a particular database has used to index articles. These MeSH terms may differ depending on the databases. Thus, I have thoroughly searched the appropriate MeSH terms for our three main concepts and described each databases’ MeSH terms below.* - ***MeSh subject headings*** *have either been Exploded (DE) or searched as a Major Concept (MM) relating to its relevance.* - ***(ti,ab)*** *searches the term in title/abstract.* - ***(*)*** *command at the end of the keyword or part of the keyword retrieves unlimited suffix variations (e.g., mindful* for mindfulness, mindfulness-based intervention* | |  |

Supplementary Table 3.

*Search Strategy Medline*

| ***MEDLINE*** *26/04/2021* | |  |
| --- | --- | --- |
| **#** | **Search Terms** | **Results** |
| **Concept 1: Substance** | | |
| **#1** | (MM "Drug Misuse+") OR (MM "Drug Users") OR (MM "Substance-Related Disorders+") OR (MM "Substance Abuse, Oral") OR (MM "Substance Abuse, Intravenous") OR (MM "Opioid-Related Disorders") OR (MM "Heroin Dependence") OR (MM "Heroin") OR (MM "Alcoholism") OR (MM "Alcoholics") OR (MM "Cocaine") OR (MM "Crack Cocaine") OR (MM "Methamphetamine") OR (MM "Amphetamine") OR (MM "Amphetamine-Related Disorders") OR (MM "Cannabis") OR (MM "Marijuana Abuse") OR (MM "Tobacco Use Disorder") OR (MM "Narcotic-Related Disorders") | **262,074** |
| **#2** | drug* OR substance* OR narcotic* OR addict* OR heroin* OR opiate* OR opioid* OR opium OR alcohol* OR cocaine* OR “crack cocaine” OR methamphetamine* OR amphetamine* OR ice* OR ecstasy OR MDMA OR cannabis* OR marijuana* OR tobacco* OR nicotin* OR hallucinogen* OR ketamine OR LSD OR psilocybin OR DMT OR psychotropic OR polydrug* OR polysubstance* OR kratom OR prescription* OR “gamma hydroxybutyrate” OR GHB OR GBH; ti,ab | **7,483,718** |
| **#3** | #1 OR #2 | **7,484,566** |
| **Concept 2: Functional Neuroimaging** | | |
| **#4** | (MM "Functional Neuroimaging+") OR (MM "Neuroimaging") | **43,997** |
| **#5** | fMRI OR functional MRI OR functional magnetic resonance imag* OR functional neuroimag* OR BOLD OR blood oxygen level-dependent OR brain function* OR brain connect* OR neural connect* OR functional connect* OR brain activity OR neural activity OR functional activity; ti,ab | **846,309** |
| **#6** | #4 OR #5 | **858,712** |
| **Concept 3: Mindfulness** | | |
| **#7** | (MM "Mindfulness") OR (MM "Mindfulness-Based Interventions”) OR (MM "Meditation”) | **6,263** |
| **#8** | mindful* OR meditat* OR MBI OR MBSR OR MBCT OR MBRP; ti,ab | **22,272** |
| **#9** | #7 OR #8 | **22,272** |
| **#10** | #3 **AND** #6 **AND** #9 | **198** |

| ***CINAHL*** *26/04/2021* | |  | |  |
| --- | --- | --- | --- | --- |
| **#** | **Search Terms** | | **Results** | |
| **Concept 1: Substance** | | | |  |
| **#1** | (MM "Substance Use Disorders") OR (MM "Substance Dependence+") OR (MM "Substance Abuse") OR (MM "Drugs, Non-Prescription") OR (MM "Narcotics") OR (MM "Street Drugs") OR (MM "Heroin") OR (MM "Alcoholism") OR (MM "Cocaine") OR (MM "Crack Cocaine") OR (MM "Amphetamine") OR (MM "Methylenedioxymethamphetamine") OR (MM "Cannabis") OR (MM "Nicotine") | | **126,042** | |
| **#2** | drug* OR substance* OR narcotic* OR addict* OR heroin* OR opiate* OR opioid* OR opium OR alcohol* OR cocaine* OR “crack cocaine” OR methamphetamine* OR amphetamine* OR ice* OR ecstasy OR MDMA OR cannabis* OR marijuana* OR tobacco* OR nicotin* OR hallucinogen* OR ketamine OR LSD OR psilocybin OR DMT OR psychotropic OR polydrug* OR polysubstance* OR kratom OR prescription* OR “gamma hydroxybutyrate” OR GHB OR GBH; ti,ab | | **1,258,850** | |
| **#3** | #1 OR #2 | | **1,272,771** | |
| **Concept 2: Functional Neuroimaging** | | | |  |
| **#4** | fMRI OR functional MRI OR functional magnetic resonance imag* OR functional neuroimag* OR BOLD OR blood oxygen level-dependent OR brain function* OR brain connect* OR neural connect* OR functional connect* OR brain activity OR neural activity OR functional activity; ti,ab | | **81,652** | |
| **Concept 3: Mindfulness** | | | |  |
| **#5** | (MM "Mindfulness") OR (MM "Meditation") | | **6,508** | |
| **#6** | mindful* OR meditat* OR MBI* OR MBSR OR MBCT OR MBRP | | **19,075** | |
| **#7** | #5 OR #6 | | **19,075** | |
| **#8** | #3 **AND** #4 **AND** #7 | | **42** | |

Supplementary Table 4.

*Search Strategy CINAHL*

| Supplementary Table 5.  *Search Strategy Web of Science*  ***Web of Science*** *– 26/04/2021* | |  |
| --- | --- | --- |
| **#** | **Search Terms** | **Results** |
| **Concept 1: Substance** | | |
| **#1** | TS=(drug* OR substance* OR narcotic* OR addict* OR heroin* OR opiate* OR opioid* OR opium OR alcohol* OR cocaine* OR “crack cocaine” OR methamphetamine* OR amphetamine* OR ice* OR ecstasy OR MDMA OR cannabis* OR marijuana* OR tobacco* OR nicotin* OR hallucinogen* OR ketamine OR LSD OR psilocybin OR DMT OR psychotropic OR polydrug* OR polysubstance* OR kratom OR prescription* OR “gamma hydroxybutyrate” OR GHB OR GBH)  Indexes=SCI-EXPANDED, SSCI, ESCI Timespan=1900-2021 | [**3,817,084**](https://apps-webofknowledge-com.ezproxy1.acu.edu.au/summary.do?product=WOS&doc=1&qid=6&SID=C6kNKphVx7fhYiaYknH&search_mode=AdvancedSearch&update_back2search_link_param=yes) |
| **Concept 2: Functional Neuroimaging** | | |
| **#2** | TS=(fMRI OR functional MRI OR functional magnetic resonance imag* OR functional neuroimag* OR BOLD OR blood oxygen level dependent OR brain function* OR brain connect* OR neural connect* OR functional connect* OR brain activity OR neural activity OR functional activity)  Indexes=SCI-EXPANDED, SSCI, ESCI Timespan=1900-2021 | **1,170,521** |
| **Concept 3: Mindfulness** | | |
| **#3** | TS=(mindful* OR meditat* OR mbi* OR mbsr OR mbct OR mbrp)  Indexes=SCI-EXPANDED, SSCI, ESCI Timespan=1900-2021 | **53,476** |
| **#4** | #1 AND #2 AND #3 | **165** |

Supplementary Table 6.

*Search Strategy Scopus*

| ***Scopus*** *– 26/04/2021* | |  |
| --- | --- | --- |
| **#** | **Search Terms** | **Results** |
| **Concept 1: Substance** | | |
| **#1** | TITLE-ABS-KEY(drug* OR substance* OR narcotic* OR addict* OR heroin* OR opiate* OR opioid* OR opium OR alcohol* OR cocaine* OR “crack cocaine” OR methamphetamine* OR amphetamine* OR ice* OR ecstasy OR MDMA OR cannabis* OR marijuana* OR tobacco* OR nicotin* OR hallucinogen* OR ketamine OR LSD OR psilocybin OR DMT OR psychotropic OR polydrug* OR polysubstance* OR kratom OR prescription* OR “gamma hydroxybutyrate” OR GHB OR GBH) | **10,914,972** |
| **Concept 2: Functional Neuroimaging** | | |
| **#2** | TITLE-ABS-KEY("fMRI" OR "functional magnetic resonance imaging" OR "functional MRI" OR (("brain imaging" OR neuroimag* OR BOLD OR "blood oxygen level-dependent" OR connectivity OR activity) AND functional)) | **606,247** |
| **Concept 3: Mindfulness** | | |
| **#3** | TITLE-ABS-KEY(*mindful**  OR  *meditat**  OR  *mbi**  OR  *mbsr*  OR  *mbct*  OR  *mbrp*) | **61,519** |
| **#4** | #1 AND #2 AND #3 | **129** |

Supplementary Table 7.

*Search Strategy PubMed*

| ***PubMed*** *– 26/04/2021* | |  |
| --- | --- | --- |
| **#** | **Search Terms** | **Results** |
| **Concept 1: Substance** | | |
| **#1** | drug*[Text Word] OR substance*[Text Word] OR narcotic*[Text Word] OR addict*[Text Word] OR heroin*[Text Word] OR opiate*[Text Word] OR opioid*[Text Word] OR opium[Text Word] OR alcohol*[Text Word] OR cocaine*[Text Word] OR "crack cocaine"[Text Word] OR methamphetamine*[Text Word] OR amphetamine*[Text Word] OR ice*[Text Word] OR ecstasy[Text Word] OR MDMA[Text Word] OR cannabis*[Text Word] OR marijuana*[Text Word] OR tobacco*[Text Word] OR nicotin*[Text Word] OR hallucinogen*[Text Word] OR ketamine[Text Word] OR LSD[Text Word] OR psilocybin[Text Word] OR DMT[Text Word] OR psychotropic[Text Word] OR polydrug*[Text Word] OR polysubstance*[Text Word] OR kratom[Text Word] OR prescription*[Text Word] OR "gamma hydroxybutyrate"[Text Word] OR GHB[Text Word] OR GBH[Text Word] | **7,236,178** |
| **Concept 2: Functional Neuroimaging** | | |
| **#2** | (("fMRI"[Text Word] OR "functional magnetic resonance imaging"[Text Word] OR "functional MRI"[Text Word]) OR ("brain imaging"[Text Word] OR neuroimag* OR BOLD OR "blood oxygen level-dependent"[Text Word] OR connectivity OR activity)) AND (functional[Text Word]) | **573,986** |
| **Concept 3: Mindfulness** | | |
| **#3** | mindful*[Text Word] OR meditat*[Text Word] OR mbi*[Text Word] OR mbsr[Text Word] OR mbct[Text Word] OR mbrp[Text Word] | **21,506** |
| **#4** | #1 AND #2 AND #3 | **82** |

Supplementary Figure 1.

*PRISMA Flowchart*

**Records identified through database searching**
(*n* = 735)

PsycInfo (*n* = 119)

MEDLINE (*n* = 198)

CINAHL (*n* = 42)

Web of Science (*n* = 165)

Scopus (*n = 129)*

PubMed (*n* = 82)

## **Identification**

**Records after duplicates removed**(*n* = 451)

## **Screening**

**Records excluded**(*n* = 436)

Reasons?

**Titles/abstracts screened**(*n* = 451)

**Full-text articles excluded**
(*n* = 8)

Interventions other than MBI, or no intervention (*n* = 1)

Imaging techniques other than fMRI (*n* = 5)

Comorbid mental health disorders (*n* = 1)

Reviews/protocols (*n* = 1)

**Full-text articles screened**(*n* = 15)

## **Eligibility**

## **Included**

**Selected studies**(*n* = 7)

*Abbreviations:* MBI, mindfulness-based intervention; fMRI, functional magnetic resonance imaging.

Supplementary Text 1.

*1.1 Sample socio-demographic characteristics*

Three out of the seven studies reported participants handedness. One study contained only right-handed participants, while two studies included participants of any handedness. Five studies included mostly males. The sample size was on average 36 participants (range: n=5 to 67). All studies were conducted in the USA, with one exceptions (1) (Egypt). In four studies, samples were recruited through advertisement in the general community (2-5). The other three studies recruited participants via university campuses (6, 7) or an addiction inpatient unit (1).

*1.2 Overview of substance use and misuse*

*1.2.1 Minimum Substance Use levels*

All studies set a minimum threshold for determining inclusion in the substance use groups. All studies of nicotine users required consumption of >10 cigarettes/day. One study of opiate users required meeting DSM-5 diagnostic criteria of opiate dependence (1).

*1.2.2 Measurement and level of substance exposure*

Substance exposure levels were measured via frequency of use in all but one study (1), and duration of use in 3 studies (1, 2, 7).

The samples with nicotine dependence comprised heavy daily smokers (average duration of 23 years; range 17-to-26 years), with a mean consumption of 16 daily cigarettes (range: 10-to-22 daily cigarettes). The sample of opiate users (mostly heroin) had a duration of use of 10 years.

*1.2.3 Overview of measurement and reporting of substance use disorders*

The severity of problems with substance use was measured using screening tools with diagnostic cut-offs or full diagnostic tools (e.g., diagnostic interviews) or both in five studies. Therefore, all samples had likely substance dependence, henceforth termed SUD for simplicity. Four of six studies used the Fagerstrom Test for Nicotine Dependence (FTND) to determine severity of nicotine-dependence alongside participants reporting smoking >10 cigarettes per day (2, 5-7). In two studies participants self-reported smoking > 10 cigarettes per day only with no other measure of dependence employed (3, 4). Opiate dependence was confirmed using patient history, the Structured Clinical Interview for DSM‑1 Disorder (SCID-1) (1), positive urine results and the Addiction Severity Index (ASI) (1).

*1.2.4 Motivation to quit*

Participants’ motivation to quit was measured in all but two studies (2, 5). All of the participants in the opiate dependence study expressed interest in quitting (1), as well as half of the samples with nicotine dependence (1, 3, 4, 7). One study excluded nicotine dependent participants who wanted to quit (6).

*1.2.5 Minimum Abstinence Duration*

Of the seven studies, three required participants to abstain from substance use. These included 20+ days of abstinence from opiates at baseline (1), and abstinence of nicotine needed for 12-hours before baseline and follow-up testing .

*1.2.6 Toxicology analyses confirming substance exposure and abstinence.*

Two out seven studies performed toxicology analyses from specimens to confirm substance exposure and/or abstinence. Of these, one study utilised urine toxicology to measure exposure to any other psychoactive substance besides nicotine (7), and the study of opioid users utilised urine toxicology to measure presence vs absence of opiate metabolites to confirm 20-day abstinence required to start the MBI.

The five of the six studies of cigarette users utilised smokerlysers (i.e., a instrument akin the breathalyser for nicotine readings) at baseline and follow-up assessments to confirm presence vs absence of measure the level of carboxy-hemoglobin (%COHb) concentration which is a proxy for *carbon dioxide* in lungs and blood. This percentage converts to the actual number of cigarettes participants consumed that day, which is reported for five studies.

*1.3 Exclusion Criteria*

*1.3.1 Mental Health and Neurological Disorders*

Six of the seven studies examined samples with a SUD but excluded samples with a confirmed dual diagnosis of SUD and an axis-1 mental health disorder (e.g., major depression, anxiety, psychosis), and excluded people with neurological disorders (e.g., brain injury, dementia, stroke), and major medical conditions (e.g., epilepsy). One study did not explain this exclusion criterion.

*1.3.2 Medication.*

Four of the seven studies excluded participants taking psychotropic medications that affect the central nervous system. One study excluded a blanket of all medical contraindications to fMRI. In addition, two studies did not explain this exclusion criterion.

*1.3.3 Substance Use Exposure.*

All six of the nicotine studies excluded participants with co-current substance dependency beyond nicotine, or if participants met DSM-IV criteria for substance use disorder in the past year, or the past month. The opiate study did not explain this exclusion criterion.

*1.4 Allocation to condition*

Of the four studies which entailed some form of random allocation to intervention conditions, three included a randomised allocation procedure, and one entailed a pseudo-randomised procedure. In this study, participants were recruited for a MORE intervention group first, and then other participants were selected for the control group based on socio-demographic variables (e.g. age, gender, race) to match the intervention group (2).

*1.5 MRI Characteristics*

The most frequently used MRI scanner brands were Siemens (*n*=3), followed by Phillips (*n*=2). The strength of the MRI scanners ranged from 1.5 Tesla to 3.0 Tesla.

*1.6 fMRI Characteristics*

Five studies used task-based fMRI to measure brain activity, examining blood-oxygen-level-dependent (BOLD) responses from the acquired tasks. Additionally, two studies used resting-state fMRI to measure functional connectivity identifying spontaneous fluctuations in BOLD signals while the brain is “at rest" “(e.g., absence of a task).

Supplementary Textbox 1.

*Components of reviewed Mindfulness Based Interventions and control interventions*

| Type of intervention | Components of the intervention | Target |
| --- | --- | --- |
| *Mindfulness-based interventions* |  |  |
| Mindfulness Training | *Mindfulness training* used typical mindfulness-based strategies. Modules progressed through sessions covering items such as habit formation (i.e., instructions on how to recognise affective states and cravings and mindfully work through them practicing body scan meditation, loving-kindness meditation, and RAIN (recognise, accept, investigate, note)). Also training to identify possible triggers for habitual behaviour and acceptance of negative emotions. | Self-monitor and increase awareness (instead of reacting to) of people’s own substance use habits, triggers and cue-induced affective states. |
| Mindfulness Orientated Recovery Enhancement (MORE) | *MORE* combines mindfulness training (mindful breathing and body scan meditations), cognitive reappraisal techniques, and focusing on savouring of sensations. | MORE aims to regulate substance use behaviour, decrease negative affect and craving, and augment reward processing and positive emotions, respectively. |
| Mindfulness-based Therapy  (MBT) | *MBT* comprises different types of sessions:   1. Formal, guided group meditation sessions (e.g., mindful breathing, siting, self-inquiry, body scanning, loving kindness) and theoretical training sessions where people learnt about mindfulness and different techniques related to stress and anxiety. 2. Informal session done by individual at home (e.g., the reappraisal technique Recognize/ Acknowledge/ Investigate/Non-identify (RAIN), mindful eating, and stop stress. | MBT aims to induce changes in distress tolerance and impulsive behaviour during recovery from substance dependence. |
| Integrative Body-Mind Training  (IBMT) | *IBMT* comprises concurrently mindfulness training, body relaxation and mental imagery while listening to soothing background music. | IBMT aims to reduce stress, increase positive emotion, and improve attention and self-control. |
| Mindful Attention task  (MA) | MA entails to actively focus on people’ own reactivity to substance-related thoughts, feelings, memories, and bodily sensations; while viewing either smoking, neutral, or aversive images and while aiming to be non-judgemental towards their own responses. | *MA* aims to train people’ mindful attention to their substance-related sensations and cravings. |
| *Control Interventions* |  |  |
| Passive viewing | Passive viewing entailed simply relaxing and looking at the picture as naturally as possible while viewing either smoking, neutral, or aversive images. |  |
| Treatment as Usual  (TAU) | *TAU* entails any regular treatments offered at the inpatient addiction facility, e.g., cognitive behavioural therapy (CBT), motivational interview, and group therapies. | Goal is treatment specific. |
| Time-control Comparison | No treatment received. | _ |
| National Cancer Institute’s QuitGuide app | The app tracks cravings, moods, slips, and smoke free progress to help build skills to remain smoke free. | It aims to help people understand their substance (tobacco) using patterns. |
| Freedom from Smoking  (FFS) | FFM is a CBT that teaches cognitive strategies to help cope with cravings, stress/negative emotions, behaviour modification, and relapse prevention. | *FFS* was developed to target smoking cessation via self-monitoring and identification of triggers. |
| Relaxation Therapy | *Relaxation therapy* entails guided sessions with physical and mental relaxation strategies e.g., progressive muscle relaxation. | Aims to focus on concentrating on the sensation of relaxation in a body part at a time. |

Supplementary Text 2.

*Intervention parameters: Mode of delivery, duration, and frequency.*

The parameters of the interventions varied across studies. The *mode of delivery* most frequently face-to-face and guided by mindfulness instructors (*n* = 4) or researcher instructions (n = 1), followed by audio recordings (*n* = 3) and smartphone apps (*n* = 2).

The interventions were delivered individually (*n* = 4), combined individual and group delivery (*n* = 2) and in groups in a single study. The duration of the interventions ranged from a single 28 min session up to eight weekly sessions with weekly practice (range: 2-to-10 times per week). All interventions were standardised for all participants (e.g., the duration and frequency of the interventions were consistent within studies). Sessions lasted from 5 minutes to 90 minutes.

Supplementary Table 8.

*Overview of sample demographic, substance use and intervention characteristics condition*

| **Author, year** | **Study design** | **Intervention** | |  | | **MBI characteristics** | | | | **MRI assessment** |
| --- | --- | --- | --- | --- | --- | --- | --- | --- | --- | --- |
|  |  | **MBI** | **Control** | **Individual/**  **group training** |  | | **Mode** | **Duration** | **Frequency** |  |
| Fahmy (1) | Randomised controlled experiment | MBT | TAU | Group | Self-directed* | | In person, in-group, audio-based instruction | 3 weeks | 5-40 mins  4 times /week | Baseline + Follow-up |
| Janes (3) | RCT | General Mindfulness Training | National Cancer Institute’s QuitGuide | Individual | Self-directed | | App-based instruction | 3 weeks | 5-15 min/day | Baseline + Follow-up |
| Kragel (5) | Within-subject | Mindfulness Training | _ | Individual | Senior mindfulness-instructor & self-directed | | In person with instructor and app-based instruction *** | 1 week | 1-hour training with instructor  + self-directed 30 min/day  + mindful eating 1/day | Baseline + Follow-up |
| Froeliger (2) | Non-RCT | MORE | Time-control | Individual & group | Researcher-instructor & self-directed | | In person instructor/audio-based instruction*** | 10 weeks | 2 hours  1/week  + 15 min/day homework | Baseline + Follow-up |
| Kober (4) | RCT | Mindfulness Training | Freedom From Smoking | Individual & group | Experienced mindfulness-instructor**  & self-directed | | In person instructor/audio-based instruction *** | 4 weeks | 90 mins  2/week  + 30 min/day homework | Follow-up |
| Tang (6) | Randomised controlled experiment | IBMT | Relaxation | Individual | Mindfulness-instructor  & self-directed | | In person instructor/audio-based instruction | 10 days | 30 mins/day | Baseline + Follow-up |
| Westbrook (7) | Within-subject | BIM | _ | Individual | Researcher-instructor | | In person instructor | Once | 28 mins | Single scan |

*Abbreviations:* IBMT, Integrative body-mind training; MBI, Mindfulness-based intervention; MBT, Mindfulness-based therapy; MORE, Mindfulness Oriented Recovery Enhancement; BIM = brief instruction in mindfulness. * group sessions where audio was delivered, no mention of a trainer asking participants about their compliance to the audio instructions. ** a single therapist with >13 year experience in mindfulness training. ***instructor-led + self-directed homework (in the form of audio or app)

Supplementary Table 9.

*Overview of time of administration of interventions and/or fMRI testing*

|  |  | Baseline | Time between Baseline and Follow-up | Follow-up |
| --- | --- | --- | --- | --- |
| Fahmy (1) | MBI |  |  |  |
|  | fMRI |  | _ |  |
| Janes (3) | MBI |  |  |  |
|  | fMRI |  | _ |  |
| Kragel (5) | MBI |  |  |  |
|  | fMRI |  | _ |  |
| Froeliger (2) | MBI |  |  |  |
|  | fMRI |  | _ |  |
| Kober (4) | MBI |  |  |  |
|  | fMRI | _ | _ |  |
| Tang (6) | MBI |  |  |  |
|  | fMRI | _ | _ |  |
| Westbrook (7) | MBI |  | _ | _ |
|  | fMRI |  | _ | _ |

*Abbreviations:* fMRI, functional magnetic resonance imaging; MBI, Mindfulness-based intervention;

Supplementary Table 10.

*Overview of neurobehavioral changes pre-to-post control intervention in substance use disorders*

| **Author, Year** | **Control** | **Behaviour changes pre-post *control* intervention** | | **fMRI** | | | **Brain functional changes** | | | | **Brain-behaviour associations** |
| --- | --- | --- | --- | --- | --- | --- | --- | --- | --- | --- | --- |
|  |  | **Substance use** | **Mindfulness/other** | **Task** | **Analysis method** | **Function** | **Direction, region** | **Cohen’s *d*** | ***p*, correction** | **Z or t** |  |
| Fahmy (1) | TAU | _ | 🡩 mindfulness (FMI), tolerance, regulation (DTS)  🡫 sensation seeking (UPPS-P)  = DTS (appraisal, absorption), UPPS-P (positive urgency, negative urgency, premeditation, perseverance) | Rest  (eyes closed) | Whole brain  ICA  (aDMN, pDMN) | Connectivity | 🡫 Hippocampus, precentral, thalamus, pallidum (aDMN/pDMN)  🡩 Sup/mid temporal gyrus (aDMN) | _ | P<.05  FDR-corrected | Z=3.5-4.7 | *N.S. Cor.* FMI, DTS, UPPS-P & connectivity within aDMN/pDMN |
| Janes (3) | Control | 🡫 Cig/day | _ | Cue reactivity  (nicotine vs neutral) | ROI  (PCC) | Activity | = | _ | P<.05  corrected with permutation testing | _ | *N.S. Cor.* ΔCigs/day & PCC, mPFC and anterior insula |
| Kober (4)^*^ | FFS | 🡩 Craving  post stress scenarios | 🡩 stress post stress scenarios (craving & stress rating before each stressful / neutral scenario in the MRI task) | Cue reactivity  (stress vs neutral) | Whole brain | Activity | = | _ | P<.05 FWE-corrected | _ | _ |
| Tang (6) | Relaxation | = craving, CO |  | Rest  (eyes closed) | Whole brain  (fALFF) | Connectivity | = | _ | P<.05  montecarlo -corrected | _ | _ |

*^*^Cross-sectionally, post intervention*

🡩 Increased; 🡫 Decreased; Δ Change.

*Abbreviations:* aDMN, anterior default mode network; CO, carbon monoxide; Cor, correlation; DTS, distress tolerance scales; fALFF, fractional amplitude of low-frequency fluctuations; FFS, freedom from smoking; FMI, Freiburg mindfulness inventory; ICA, independent component analysis; MBI, mindfulness-based intervention; mPFC, medial prefrontal cortex; MRI, magnetic resonance imaging; N.S., non-significant; PCC, posterior cingulate cortex; pDMN, posterior default mode network; ROI, region of interest; Sup, superior; TAU, treatment as usual; UPPS-P, impulsive behaviour scale.

Supplementary Text 3.

*Risk of Bias and Quality Assessment*

We assessed the risk of bias (low, moderate, high bias) in relation to the evaluation of the interventions and of the fMRI methodology, using two distinct tools (*Supplementary Tables 11-12*). The first tool was used in a previous study to assess bias in relation to (reporting about) the *intervention* aspects of study design (8) against 14 criteria (*Supplementary Table 11*). They included the reporting of: research objectives; method of recruitment and randomisation procedures; presence of a control group; study design (e.g., RCT, randomised controlled design, random allocation of participants to groups, within-subject design); concealment of treatment allocation; whether group characteristics were significantly different at baseline; number of drop outs and related reasons; how mindfulness practice was documented; qualifications and training of mindfulness teachers; checks on intervention fidelity; and reporting of participant class attendance/completion of modules (i.e., presence vs absence)*.* Within each study, we rated the risk of bias as *low* when all quality criteria or all criteria, but one was endorsed; *moderate* when all but 2-3 quality criteria were endorsed; and *high* when 4+ quality criteria were not met. To assess the risk of bias for each criterion across studies we computed the average score across studies. A mean score closer to 0 indicated a low risk of bias, and closer to 1 indicated a high risk of bias.

The second tool measured the quality of reporting of fMRI results using 11 criteria based on standardized reporting (9), shown in *Supplementary Table 12*. They ranked the reporting of type of fMRI design; sample handedness and gender; software analysis package; reasons for scan rejection; volumes acquired per session; method for motion correction and multiple comparison correction; type of correction applied; and descriptions of the first and second level contrasts. Possible study scores ranged from 0 or 1 for each item. Within each study an overall score of 0-7 indicated *poor quality*, an overall score of 8-9 indicated *moderate quality*, and an overall score of 10-11 indicated *high quality*. Across all studies, an overall mean score of 0-3 indicated *low quality,* an overall mean score of 4-5 indicated *moderate quality,* and an overall mean score of 6-7 indicated *high quality.* The average score was computed across all studies. A score closer to 0 indicated poor quality reporting, and a score closer to 1 indicated high quality reporting.

*Risk of Bias: Study Design and Intervention*

As shown in *Supplementary Table 11*, the reviewed literature overall had on average a moderate risk of bias across 14 criteria (0.32, on a scale ranging from low risk of bias = 0, to high risk of bias =1) (8) critical appraisal tool for assessing intervention quality across 14 criteria.

First, overall, the included studies had *low* *risk of bias* in relation to 5 criteria, namely, *research objectives, recruitment procedure, the number of dropouts reported, reason for dropout stated* and the use of a *comparison group* (i.e., the latter 2 criteria were endorsed by all studies but one).

Second, overall the studies had a *moderate risk of bias* in relation to 8 criteria: (i) *study design* (i.e., which ranged from RCTs (n = 4), within subject designs (n = 2), and a non-randomized control trial); (ii)-(iii) reporting of *trial* *randomisation* *and randomisation procedures* (i.e. rated as being met by 4 of 7 studies); (iv) intervention *groups were similar at baseline,* for all but two studies; (v) reporting of *how mindfulness practice was recorded*, (vi) reporting of whether mindfulness *teachers underwent training*, (vii) used a scale to check *intervention adherence*, and (viii) *reporting of class attendance*. Finally, the *risk of bias* was high for the criterion *treatment allocation*, as no study concealed this (with one exception).

Within individual studies, the risk of bias ranged from low (n=2 studies met all but 1 criteria), to moderate (2 studies met 2-to-3 criteria) to high (i.e., 3 studies failed to meet 9 or less of the 14 criteria), with an average score of 2.71 indicating an overall *moderate risk of bias*.

*Assessment of Quality of the fMRI methodologies*

As shown in *Supplementary Table 12*, the quality of the reporting in relation to fMRI methodologies was on average high against 11 criteria (0.86, on a scale from poor quality reporting = 0, to high quality reporting = 1) stipulated in the standardized reporting guidelines for fMRI quality (9).

The studies were rated high on quality for 7 criteria. Specifically, all studies reported: the *type of fMRI design*, *software package* used to analyze the data, methods used for *motion correction*, method used to *control for multiple comparisons*, which *type of correction* was used to control for multiple comparison, the types of *first level contrasts* used and participants’ *sex*.

The reviewed studies were rated moderate in quality for 4 criteria, as all but two studies reported *scan rejection reasons* and *described the second level contrasts*, and only 4 studies reported the *number of volumes per session*. Finally, only one criterion was ranked as poor quality with only 3 studies reporting participants’ *handedness*.

Across individual studies, the quality ranged from high for all, except for two studies of moderate quality and one of low quality, with an average score of 9.43 indicating overall *high quality of reporting*.

Supplementary Table 11.

*Overview of* *Risk of Bias of the reviewed functional neuroimaging studies of mindfulness-based interventions in substance use disorder, using the tool developed by Young and colleagues (2018)*

| Author, year | Research objective described? | Recruitment procedure described? | Study design | Comparison group | Was the trial randomised? | Randomisation described? | Treatment allocation concealed? | Groups similar at baseline? | N dropouts  reported? | Dropout reasons stated? | How was mindfulness practice recorded? | Teacher training reported? | Scale used to check intervention adherence? | Class attendance/module reported? | **Within-study Risk of bias** |
| --- | --- | --- | --- | --- | --- | --- | --- | --- | --- | --- | --- | --- | --- | --- | --- |
| **Average risk of bias [low = 0 = high = 1]** | 0 | 0 | 0.43 | 0.14 | 0.43 | 0.43 | 0.86 | 0.33 | 0 | 0.14 | 0.43 | 0.43 | 0.43 | 0.43 | **0.32** |
| Fahmy (1) | 0 | 0 | random group allocation | 0 | 0 | 0 | 1 | 0 | 0 | 0 | Daily-self report form | 1 | 1 | 1 | 4 |
| Janes (3) | 0 | 0 | RCT | 0 | 0 | 0 | 0 | 0 | 0 | 0 | Daily-self report form | N/A | 0 | 0 | 0 |
| Kragel (5) | 0 | 0 | within-subject | 1 | 1 | N/A | 1 | N/A | 0 | 1 | Daily-self report form | 0 | 0 | 0 | 4 |
| Froeliger (2) | 0 | 0 | Non-RCT | 0 | 1 | N/A | 1 | 0 | 0 | 0 | Daily-self report form | 0 | 1 | 1 | 4 |
| Kober (4) | 0 | 0 | RCT | 0 | 0 | 0 | 1 | 0 | 0 | 0 | Not specified | 0 | 1 | 1 | 3 |
| Tang (6) | 0 | 0 | randomise controlled design | 0 | 0 | 0 | 1 | 0 | 0 | 0 | Not specified | 0 | 0 | 0 | 1 |
| Westbrook (7) | 0 | 0 | within-subject | 0* | 1 | N/A | 1 | N/A | 0 | 0 | Not specified | 1 | 0 | 0 | 3 |
|  |  |  |  |  |  |  |  |  |  |  |  |  |  | **Mean Within-study Risk of bias** | **2.71** |

*Abbreviations:* 0, low risk; 1, high risk; RCT, randomized control trial; N/A, not applicable; * Within-subject comparison group

Supplementary Table 12.

*Quality Assessment of the reviewed functional neuroimaging studies of mindfulness-based interventions in substance use disorder*

| Author, year | Type of fMRI design | Handedness | Sex reported | Scan rejection reason | Volumes/session | Software package specified | Motion correction method | Multiple comparison correction method | Type of correction | First level contrasts described | Second level contrasts described | Quality score |
| --- | --- | --- | --- | --- | --- | --- | --- | --- | --- | --- | --- | --- |
| **Average reporting, from poor = 0 and high = 1** | 1 | 0.43 | 1 | 0.71 | 0.57 | 1 | 1 | 1 | 1 | 1 | 0.71 | **0.86** |
| (1) | Rest | 1 | 1 | 1 | 1 | 1 | 1 | 1 | Cluster | 1 | 1 | 11 |
| Janes (3) | Task | 0 | 1 | 1 | 0 | 1 | 1 | 1 | Cluster | 1 | 1 | 9 |
| Kragel (5) | Task | 0 | 1 | 0 | 0 | 1 | 1 | 1 | Cluster | 1 | 0 | 7 |
| Froeliger (2) | Rest/Task | 1 | 1 | 1 | 1 | 1 | 1 | 1 | Cluster | 1 | 1 | 11 |
| Kober (4) | Task | 0 | 1 | 1 | 1 | 1 | 1 | 1 | Voxel-wise | 1 | 1 | 10 |
| Tang (6) | Rest | 0 | 1 | 1 | 0 | 1 | 1 | 1 | Cluster | 1 | 0 | 8 |
| Westbrook (7) | Task | 1 | 1 | 0 | 1 | 1 | 1 | 1 | Voxel-wise | 1 | 1 | 10 |
|  |  |  |  |  |  |  |  |  |  |  | **Mean within-study quality score** | **9.43** |

*Abbreviations:* 1, not reported; 0, reported

**References**

1. Fahmy R, Wasfi M, Mamdouh R, Moussa K, Wahba A, Schmitgen MM, et al. Mindfulness-based therapy modulates default-mode network connectivity in patients with opioid dependence. European Neuropsychopharmacology. 2019;29(5):662-71.

2. Froeliger B, Mathew A, McConnell P, Eichberg C, Saladin M, Carpenter M, et al. Restructuring reward mechanisms in nicotine addiction: a pilot fMRI study of mindfulness-oriented recovery enhancement for cigarette smokers. Evidence-Based Complementary and Alternative Medicine. 2017;2017:7018014.

3. Janes AC, Datko M, Roy A, Barton B, Druker S, Neal C, et al. Quitting starts in the brain: a randomised controlled trial of app-based mindfulness shows decreases in neural responses to smoking cues that predict reductions in smoking. Neuropsychopharmacology. 2019;44(9):1631-8.

4. Kober H, Brewer JA, Height KL, Sinha R. Neural stress reactivity relates to smoking outcomes and differentiates between mindfulness and cognitive-behavioral treatments. NeuroImage. 2017;151:4-13.

5. Kragel EA, Sweitzer MM, Davis JM. The Effect of Brief Mindfulness Training on Brain Reactivity to Food Cues During Nicotine Withdrawal: A Pilot Functional Imaging Study. Mindfulness. 2019;10(11):2272-6.

6. Tang YY, Tang R, Posner MI. Brief meditation training induces smoking reduction. Proceedings of the National Academy of Sciences. 2013;110(34):13971-5.

7. Westbrook C, Creswell JD, Tabibnia G, Juson E, Kober H, Tindle HA. Mindful attention reduces neural and self-reported cue-induced craving in smokers. Social Cognitive and Affective Neuroscience. 2013;8(1):73-84.

8. Young KS, van der Velden AM, Craske MG, Pallesen KJ, Fjorback L, Roepstorff A, et al. The impact of mindfulness-based interventions on brain activity: A systematic review of functional magnetic resonance imaging studies. Neuroscience & Biobehavioral Reviews. 2018;84:424-33.

9. Poldrack RA, Fletcher PC, Henson RN, Worsley KJ, Brett M, Nichols TE. Guidelines for reporting an fMRI study. NeuroImage. 2008;40(2):409-14.
